# Inequalities in health system coverage and quality: a cross-sectional survey of four Latin American countries

**DOI:** 10.1016/S2214-109X(23)00488-6

**Published:** 2023-12-11

**Authors:** Javier Roberti, Hannah H Leslie, Svetlana V Doubova, Jesús Medina Ranilla, Agustina Mazzoni, Laura Espinoza, Renzo Calderón, Catherine Arsenault, Ezequiel García-Elorrio, Patricia J García

**Affiliations:** a Institute for Clinical Effectiveness and Health Policy, Buenos Aires, Argentina; b Epidemiology and Public Health Research Centre, National Council for Scientific and Technical Research, Buenos Aires, Argentina; c Division of Prevention Science, Department of Medicine, University of California San Francisco, San Francisco, CA, USA; d Epidemiology and Health Services Research Unit, Mexican Institute of Social Security, Mexico City, Mexico; e School of Public Health, Cayetano Heredia University, Lima, Peru; f Department of Epidemiology, Biostatistics and Occupational Health, McGill University, Montreal, QC, Canada; g Department of Global Health, Milken Institute School of Public Health, George Washington University, Washington, DC, USA

## Abstract

The premise of health as a human right in Latin America has been challenged by health system fragmentation, quality gaps, a growing burden of chronic disease, sociopolitical upheaval, and the COVID-19 pandemic. We characterised inequities in health system quality in Colombia, Mexico, Peru, and Uruguay. We did a cross-sectional telephone survey with up to 1250 adults in each country. We created binary outcomes in coverage, user experience, system competence, and confidence in the system and calculated the slope index of inequality by income and education. Although access to care was high, only a third of respondents reported having a high-quality source of care and 25% of those with mental health needs had those needs met. Two-thirds of adults were able to access relevant preventive care and 42% of older adults were screened for cardiovascular disease. Telehealth access, communication and autonomy in most recent visit, reasonable waiting times, and receiving preventive health checks showed inequalities favouring people with a high income. In Uruguay, inequality between government and social security services explained a substantial proportion of disparities in preventive health access. In other study countries, inequalities were also substantial within government and social security subsectors. Essential health system functions are unequal in these four Latin American countries.

This is the fifth in a **Series** of six papers about the People's Voice Survey on health system performance. All papers in the Series are available at www.thelancet.com/series/peoples-voice-survey

## Background

Latin America is characterised by substantial socioeconomic inequalities between and within countries,^1^ affecting health-care delivery, access to health services, and population health outcomes.^2^, ^3^ Inequities in health coverage and outcomes persist despite a consistent focus on the right to health, reforms to strengthen health coverage in the 1990s, and commitments to universal health coverage.^3^, ^4^, ^5^ Moreover, corruption and political upheaval in the region have further weakened effective delivery of health care, contributing to persistent barriers to high-quality care for substantial proportions of individuals, especially low-income groups or those with public coverage.^2^, ^6^, ^7^, ^8^, ^9^, ^10^ Populations with inadequate access to equitable health systems are burdened with a higher prevalence of morbidity and mortality from non-communicable diseases, which are the leading cause of death in the region.^11^, ^12^ Existing inequalities are expected to be further accentuated by the rapid ageing of the population.^11^, ^12^ Furthermore, the COVID-19 pandemic has shown the vulnerability of health systems in the region; mounting evidence indicates the impact of the pandemic on preventive care access and timely and continuous care.^13^, ^14^, ^15^, ^16^, ^17^

Although substantial research has considered inequities in health coverage within Latin America, there has been little attention given to people-centred measures of health-care quality nationally.^1^, ^3^, ^18^, ^19^ In fact, existing and previous surveys of health systems are largely one-time and single country studies with a focus on coverage and on patient satisfaction.^20^ Little research exists on socioeconomic inequalities in health-care quality in Latin America.

Health sectors in most Latin American countries are characterised by inadequate financial protection against health-care costs and fragmented service delivery—divided among government care, social security coverage for formal labour market workers, and the private sector.^1^, ^21^ Compared with government services, social security institutions typically have higher funding, larger benefit packages with no fees at the point of service, and better quality of care.^22^ Nonetheless, there is little evidence on the differences in quality by health sector type. How outcomes differ by health sector type due to disparities in service quality versus differences in the population using the distinct sectors is unclear.

**Key messages**

• Inequalities in multiple domains of health system quality were quantified, revealing income and education disparities in telehealth, user experience, and system competence in preventive care
• In all countries, at least half of respondents had preventive checks in the past year; less than 50% of older adults had complete checks for cardiovascular disease in all countries and this figure was even lower in Peru; continuity of care was higher in Uruguay than in the other countries
• Most individuals had their chronic health needs met with respect to accessing care; however, only approximately 25% of those with mental health needs received any mental health care
• Despite widespread coverage by public sources (ie, government or social security), disparities between these public subsectors and within subsectors (particularly in Colombia, Mexico, and Peru) contribute to the ongoing disparities in access to preventive care
• This cross-national assessment of various health system quality domains reveals both national differences and commonalities in the four Latin American countries, emphasising the role of health system fragmentation in continuing inequities in access and quality

With the objective of measuring the perspective of both users and non-users on the performance of health systems, the People's Voice Survey (PVS) was developed as a collaborative multinational effort and was done in Colombia, Mexico, Peru, and Uruguay. In this analysis, we describe health-care coverage and quality across the four countries, quantify inequalities in these outcomes by socioeconomic status within country, and assess the contribution of government, social security, and private health sectors to observed inequality.

## Study design and setting

This Series paper reports findings from a cross-sectional telephone survey done between July, 2022, and January, 2023, in four Latin American countries: Colombia, Mexico, Peru, and Uruguay. Key features of the health systems in these countries are described in the panel. The PVS survey instrument and methods were developed by the Quality Evidence for Health System Transformation (QuEST) Network, a global consortium for research on high-quality health systems that includes all study authors; we led implementation in Colombia, Peru, and Uruguay as part of the QuEST Latin America and Caribbean Network (QuEST LAC). The analysis is one study within the larger PVS project; appendix 1 (pp 1–13) provides information on the development process and situates these countries alongside other countries analysed elsewhere.

Panel **Health systems in studied countries**

**Colombia**

The health system is composed of two main subsectors, with 95% of the population covered: public or subsidised social insurance and contributory social insurance. The subsidised regime covers people without the ability to pay, financed through public revenues and taxes. The contributory scheme is for workers in the formal sector and dependents; in this scheme, there are people who pay additional fees for supplementary services. The rest of the population is covered by special plans (eg, armed forces, university teachers, and national oil company workers) or private plans. Service provision is not segregated by sector: coverage is administered by health insurance companies called Entidades Promotoras de Salud, most of which are private, which must offer the same benefits for the contributory and subsidised schemes. They configure the provider network through a combination of public and private clinics and hospitals.^5^

**Mexico**

The system includes heterogeneous public and private sectors. There are five social security institutions: the Mexican Institute of Social Security (IMSS) covering 54·4%, the Institute for Social Security and Services for State Workers covering 10·7%, and the medical services for oil industry workers, the Army, and the Navy collectively covering 1·9% of the population. Four bodies provide services to the population without social security: the Ministry of Health, the state health secretariats, Institute of Health for Welfare, and IMSS-Bienestar. Approximately 9·7% of the population have private health insurance.

**Peru**

The health system in Peru is fragmented. The Ministry of Health provides health services through the Comprehensive Insurance System, covering around 61% of the total population—mainly the poorest, those from the informal economy, and the unemployed and their families. The Ministry of Labour, through the Social Security System, provides health services to the working class and their families (20%). Those working in the Peruvian Armed Forces and National Police (2%) are protected by their own health institution. Private health insurance covers around 3% of the population.

**Uruguay**

The health system in Uruguay consists of the public sector (28%), social security (70%; referred to as mutuales—non-profit organisations providing services to members who are generally workers from a specific sector)—and a small private sector. The National Integrated Health System (SNIS) was created in 2007 and comprises public and private health-care providers, and is financed through the National Health Fund—a single, public and mandatory fund, with a contributory component (employers and employees) and a general state revenue component. The SNIS guarantees universal coverage through 42 eligible health providers, which provide a comprehensive package of benefits that is the same for all. The Ministry of Public Health manages the system, defines health policies, and regulates providers.^5^, ^22^

## Participants

Selection of Latin American countries for participation in the PVS was non-random based on locations of the QuEST LAC research team and QuEST affiliates and interest from country teams within the InterAmerican Development Bank. In each country, Spanish-speaking adults older than 18 years with telephone access were eligible to participate. The target sample size was a minimum of 1000 respondents per country based on the overall survey aim of obtaining population opinions about the performance of the health system by estimating the proportion of the population that agrees with a range of statements. A survey of 1000 individuals selected at random will produce an estimate within a 3% margin of error of the population proportion 95% of the time.^23^

PVS respondents were selected through random-digit dialling using a mobile phone sampling frame in Colombia, Peru, and Uruguay, and a mobile phone and landline telephone sampling frame in Mexico due to lower mobile phone coverage. Numbers identified as inactive, commercial, or roaming out of country were removed before sampling. Interviewers received training and were evaluated with pre-tests and post-tests (appendix 1 p 2). Interviewers in each country contacted the sampled numbers, with up to five attempts to reach non-responsive numbers at varying times of day; respondents reached could complete the survey at that moment or schedule a more convenient time. Surveys were completed in a single sitting following administration of informed consent.

## Survey instrument

The PVS was developed to measure health service need and use, processes of care, and confidence in the system based on the framework defined in the *Lancet Global Health* Commission on high quality health systems in the Sustainable Development Goal era.^24^ Survey items included demographics and health, health-care use and system competence, care experience, health system quality, and confidence in the health-care system. The survey is intended for biennial administration; items on care use and experience used a 12-month timeframe to capture annual care use and limit recall issues. QuEST LAC led Spanish translation, adaptation, and implementation of the survey for Colombia, Peru, and Uruguay, while the QuEST Network oversaw the PVS in Mexico (appendix 2 p 1).

The Harvard University Institutional Review Board deemed this research exempt from full review, and additional ethics approval was obtained from the ethics committees at the University Peruana Cayetano Heredia (UPCH; Study 205271) and the Central Military Hospital, Bogota, Colombia. The protocol submitted to UPCH is attached in appendix 2 (p 28).

## Measures

### Health system outcomes

The overall outcome of interest was health system quality, which was operationalised into distinct indicators within four domains: system coverage, user experience, system competence, and population confidence. We defined coverage within a high-quality health system as access to a high-quality point of care with user-centred access options and core needs met; other domains are defined directly from the *Lancet Global Health* Commission framework.^24^ Within each domain, we identified measures available in the PVS and prioritised indicators relevant to the study countries and study period (table 1). We categorised all outcomes as binary outcomes to enable calculation and comparison of inequalities across outcomes and between countries.

**Table 1:** Health system quality outcomes for the Latin American region based on the *Lancet Global Health* Commission framework

	**Indicator definition**	**Total**	**Missing**
**System coverage**			
High-quality source of care	Reports a usual source of care and rates it as very good or excellent overall quality or rates overall quality of most recent visit as very good or excellent if no usual source	4731	0
Telehealth access	Reports at least one telehealth or virtual consultation in the past 12 months	4731	5 (0·1%)
Met need for chronic condition	Among those responding yes to having any long-standing illness or health problem, reports at least one health consultation (in person or virtual) beyond any care specific to COVID-19 in the past 12 months	1389	3 (0·2%)
Met need for mental health care	Among those with less than good self-rated mental health, reports at least one interaction with a health-care professional related to mental health in the past 12 months	818	6 (0·7%)
**Processes of care: positive user experience; “Systems should also be user-focused: easy to navigate, with short waiting times and attention to people's values and preferences”**^24^			
Respect	Rating of very good or excellent for respect from health-care professionals during most recent visit (among those with an in-person visit in the past 12 months)	3946	6 (0·2%)
Communication	Rating of very good or excellent for clarity of explanation from health-care professionals during most recent visit (among those with an in-person visit in the past 12 months)	3946	7 (0·2%)
Autonomy	Rating of very good or excellent for participation in decisions regarding their own treatment during most recent visit (among those with an in-person visit in the past 12 months)	3946	20 (0·5%)
Short waiting time	Reports waiting time less than 1 hour for most recent visit	3946	37 (0·9%)
Non-discrimination	Not reporting discrimination or unequal treatment from any health professional in the past 12 months among those with at least one visit of any kind	4111	11 (0·3%)
**Processes of care: competent systems; “Competent systems provide people and communities with health promotion and prevention when healthy and effective and timely care when sick”**^24^			
Prevention: health check	Reports receiving a general health check, eye examination, or dental examination in the past 12 months	4731	0
Detection: cardiovascular disease screening	Reports a blood pressure check, blood glucose test, or cholesterol test in the past 12 months among men older than 40 years and women older than 50 years (appendix 2 pp 2–3)^25^	1939	0
Continuity	Rating of very good or excellent for provider's knowledge of health history among those with most recent visit for a chronic health-care concern	965	0
Safety	No report of experiencing a medical mistake among those with any care in the past 12 months	4111	22 (0·5%)
**Quality impacts: confidence in systems; “Confidence goes beyond the more traditional measure of satisfaction with care; it is the extent to which people trust and are willing to use healthcare”**^24^			
Quality care	Somewhat or very confident in ability to get high-quality care in case of a serious illness	4731	81 (1·7%)
Affordable care	Somewhat or very confident in ability to afford high-quality care in case of a serious illness	4731	52 (1·1%)
Responsiveness	Somewhat or very confident that public opinion is considered when making health policy	4731	62 (1·3%)
Resilience in COVID-19 pandemic	Rating of very good or excellent for government management of COVID-19 pandemic	4731	19 (0·4%)

### Population characteristics

We focused on income and education as key indicators of socioeconomic status for use in quantifying inequalities. Income was separated into eight categories in Colombia, Peru, and Uruguay and five categories in Mexico on the survey instrument; education was categorised in seven categories in all countries (appendix 2 pp 3–4). We report income in approximate tertiles and education in four categories (ie, primary or less, secondary, non-university tertiary, and university or higher) for descriptive analysis and we use all available categories for inequality analysis. We report the additional demographic characteristics of age, gender (ie, male, female, or another gender), and location (ie, rural, town or suburb, and city).

### Health system use and sectors

To compare outcomes and socioeconomic inequities across health system sectors, we classified respondents' health-care coverage and use based on the multiple sectors present in each country, including government care, social security coverage (including contributory schemes), and private coverage (appendix 2 pp 4–6). The small number of military and state oil affiliates were classified with social security as the most comparable coverage group. Given policy theoretically guaranteeing universal access to government care services in three study countries, only respondents in Peru could be considered as not covered. We further classified respondents by their source of care for usual source and most recent source, grouping into those without a source and the three sectors of care. In Colombia, where health-care delivery is integrated rather than operated separately by sector, we relied on coverage to assign users to a source of care.

## Statistical analysis

We developed this analysis plan within the broader PVS project. We used proportions to characterise the sample in each country and to quantify the key outcomes across countries. We calculated the slope index of inequality (SII) across income groups and educational attainment to quantify the absolute inequality in each outcome within each country. The SII expressed the linear difference in percentage points for each outcome between those predicted to be best off and worst off (ie, richest and poorest and most educated and least educated), assuming a linear relationship between socioeconomic rank and predicted outcome probability. We used the siilogit program in Stata to calculate the SII; siilogit calculates the relative rank on the indicator of socioeconomic status and fits an unadjusted generalised linear model with logit link for the outcome against this rank.^26^, ^27^, ^28^

To investigate the factors explaining income-related inequalities, we did a decomposition analysis. We investigated the contribution of the source of usual care (ie, none, government, social security, or private) in explaining income-related inequality for the receipt of preventive checks, an outcome with substantial income inequality across countries. First, we summarised the magnitude of inequality with a concentration index, a summary of the cumulative proportion of an outcome (ie, preventive care check) across individuals from poorest to wealthiest. We chose the Erreygers concentration index^29^ (appendix 2 p 6), which is suitable for binary outcomes and ranges from –1 (maximum pro-poor distribution) to +1 (maximum pro-wealthy distribution); 0 indicates equality.^28^, ^30^ Next, we decomposed the concentration index to quantify the contributions of individual characteristics and the health system sector of usual source of care. The contribution of each predictor is the product of the elasticity (unitless partial association of the predictor with receiving a preventive check) and the concentration index of that predictor.^31^ We reported this contribution as a percentage of the concentration index of the outcome. We decomposed observed income inequality in receiving prevention checks due to age, gender, the four education categories, location, chronic illness, and sector of usual source of care. We used the Stata program conindex for this analysis.^30^ Finally, we considered the inequality in each outcome based on source of care, with usual source of care for systemic outcomes and most recent source of care for items assessed based on most recent visit (appendix 2 p 2). We plotted outcomes by source to show between-source inequality and calculated SII within each care source to quantify within-source income inequality.

All analyses were done in Stata v17; analyses were applied to complete cases within each country with survey weights. Design weights were calculated based on telephone numbers per person. Post-stratification weights were constructed with external population statistics to weight respondents on the basis of age group, gender, location (two to five regions per country for Colombia, Peru, and Uruguay), and educational attainment; post-stratification weights were calculated within income levels for Mexican respondents. The final weight for each respondent was calculated as the product of design weight and post-stratification weight.

## Respondent characteristics

Appendix 2 (p 6) details the call outcomes for each country; 4731 surveys were completed in the four countries, with response rates of 3% in Mexico, 6% in Peru, 8% in Uruguay, and 13% in Colombia (appendix 1, pp 9–10). Respondents were older in Uruguay (table 2) and twice as likely to live in a city as respondents in Mexico. Respondents in Mexico and Peru were concentrated in the low-income (lowest approximate tertile) categories. Responses to demographic items were generally complete, except for 7·8% of respondents declining to report an income category in Mexico. Government health coverage was the most common type in Colombia (54·7%) and Peru (50·1%), whereas social security schemes covered half of respondents in Mexico and Uruguay. A sixth of respondents in Peru reported no current coverage. Despite low private coverage, use of private facilities accounted for close to 20% of respondents in Mexico and Peru and 9·3% in Uruguay; use was not distinguished between public and private sources in Colombia due to integration of care delivery. Health service use was high: most respondents had a usual source of care or a visit in the past 12 months; frequent users (>4 visits) consisted of a third of respondents in Colombia, Mexico, and Peru and half of respondents in Uruguay. Respondents in Uruguay also had the highest levels of self-reported chronic illness (43·2% compared with roughly 25% in other countries). Nearly 25% of respondents in Mexico and Peru reported a mental health concern.

**Table 2:** Demographics

	**Colombia (n=1237)**	**Mexico (n=1002)**	**Peru (n=1255)**	**Uruguay (n=1237)**
**Age (years)**				
18–29	28·3%	27·8%	26·1%	21·5%
30–39	21·1%	21·2%	23·1%	20·1%
40–49	16·3%	18·9%	19·0%	16·3%
50–59	15·6%	14·5%	13·7%	15·1%
≥60	18·8%	17·6%	18·1%	26·8%
Missing	0	0	0	0·1%
Median (IQR)	40 (28–55)	40 (28–55)	40 (29–54)	45 (32–60)
**Gender**				
Male	47·9%	47·1%	49·9%	47·7%
Female	51·8%	52·5%	49·9%	52·0%
Another gender	0·1%	0·3%	0·1%	0·3%
Missing	0·2%	0	0·1%	0
**Location**				
Rural	14·1%	21·8%	17·7%	7·5%
Town or suburb	27·4%	36·8%	17·5%	10·5%
City	58·2%	40·3%	64·8%	81·5%
Missing	0·3%	1·1%	0	0·6%
**Education**				
Up to primary	49·4%	29·5%	38·0%	44·2%
Secondary	29·0%	53·0%	44·8%	43·4%
Non-university tertiary	11·0%	1·6%	6·2%	2·9%
University or higher	10·5%	15·9%	10·8%	9·4%
Missing	0	0	0·2%	0·1%
**Income**				
Low income	31·2%	53·6%	50·7%	44·1%
Middle income	34·0%	19·2%	32·6%	26·0%
High income	33·3%	19·4%	15·2%	28·0%
Missing	1·5%	7·8%	1·6%	1·9%
**Native language**				
Indigenous	0·3%	7·2%	14·2%	0
Spanish	97·6%	92·8%	85·8%	100·0%
Other	2·2%	0	0	0
**Health coverage**				
Uninsured	0	0	17·1%	0
Government	54·7%	38·9%	50·1%	36·5%
Social security and military	40·3%	47·8%	29·4%	56·1%
Private	3·0%	7·9%	3·2%	6·6%
Missing	2·0%	5·4%	0·1%	0·8%
**Usual source of care**				
None	21·8%	18·6%	23·8%	6·7%
Government	41·7%	25·5%	39·9%	35·9%
Social security and military	32·8%	35·3%	16·7%	48·1%
Private	2·3%	18·9%	19·5%	9·3%
Missing	1·4%	1·7%	0·1%	0
**Total health-care visits in the past 12 months**				
Non-user	13·3%	20·6%	17·4%	9·4%
Occasional user (1–4 visits)	52·6%	45·3%	49·1%	40·0%
Frequent user (>4 visits)	34·1%	34·1%	33·5%	50·5%
**Chronic illness**				
No	72·9%	76·5%	75·0%	56·8%
Yes	27·1%	23·4%	24·9%	43·2%
Missing	0	0·1%	0	0
**Mental health concern**				
No	85·0%	77·0%	73·5%	85·4%
Yes	14·7%	23·0%	26·5%	14·3%
Missing	0·3%	0	0	0·2%

Income categories: income was solicited in eight categories in Colombia, Peru, and Uruguay, and five categories in Mexico. See appendix 2 (pp 3–4) for classifications.

## Health system quality outcomes

**Figure 1:**
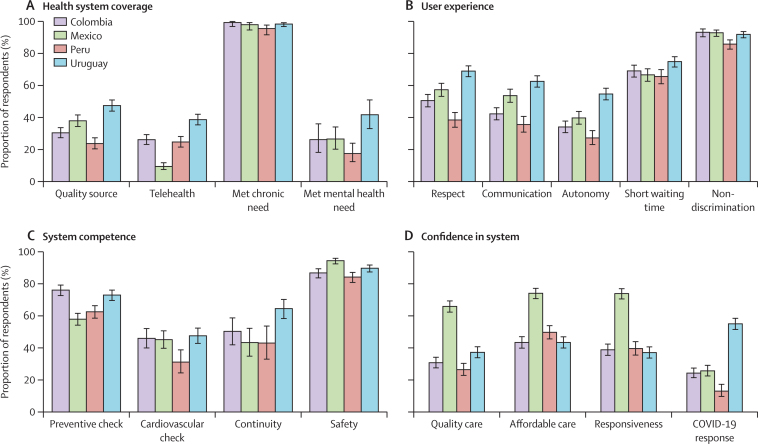
Proportion of respondents who reported health system coverage, user experience, system competence, and confidence in the health system across four Latin American countries (A) Health system coverage indicators include quality source (respondent rates usual souce or most recent source of care as very good or excellent), telehealth consultation within the past 12 months, consultation in the past 12 months among those with chronic care needs, and mental health-related consultation in the past 12 months among those with mental health care needs. (B) User experience indicators were rated very good or excellent for the most recent visit in terms of respect, communication, and autonomy, as well as waiting time under 1 h and there was no report of discrimination in health care in the past 12 months. (C) System competence indicators include receiving a preventive check in the past 12 months, cardiovascular check in the past 12 months, continuity of care (rating of very good or excellent on provider knowledge of health history among chronic care visits), and safety (no medical mistakes in past 12 months). (D) Confidence in system indicators include quality care (somewhat confident or very confident in their ability to get high-quality care in case of serious illness), affordable care (somewhat confident or very confident in their ability to afford high-quality care in case of a serious illness), responsiveness (somewhat confident or very confident that public opinion is considered when making health policy), and resilience during the COVID-19 pandemic (rating of very good or excellent for government pandemic management).

Responses regarding health system quality outcomes showed a minimal amount of missing data (<2%; table 1). In quantifying system coverage, we found that between a quarter and a half of respondents had a source they considered high quality (figure 1A). Telehealth prevalence varied from less than 10% in Mexico to nearly 40% in Uruguay. Chronic health needs were nearly universally met (at least in terms of accessing care) in all countries, whereas only a quarter of those with mental health needs received any care (17·5% in Peru and 41·7% in Uruguay). User experience ratings (figure 1B) for respect, communication, and autonomy were lowest in Peru compared with the other countries. Frequency of waiting times less than 1 h was near 70% for all countries. Non-discrimination, an outcome that should be universal within high-quality systems, was as low as 85% in Peru and roughly 93% in the other countries.

At least half of respondents reported having preventive checks in the past 12 months, with up to three-quarters in Colombia and Uruguay having preventive checks (figure 1C), although complete checks for cardiovascular disease among older adults (men aged ≥40 years and women aged ≥50 years) were less than 50% in all countries and even lower in Peru. Continuity of care was notably higher in Uruguay (64%) compared with the other countries. Medical mistakes were least commonly reported in Mexico (no mistakes reported by 94%) and most commonly reported in Peru (84% without mistakes). Reported confidence in care quality, affordability, and responsiveness exceeded half of respondents only in Mexico, where confidence in care quality and government responsiveness was nearly double the prevalence elsewhere (figure 1D). Government management of the COVID-19 pandemic was rated worst in Peru (13% favourable), poorly in Mexico and Colombia (24–26% favourable), and well in Uruguay (55% favourable).

## Socioeconomic inequities in outcomes

Within each country, income inequality occurred in multiple health system outcomes (table 3); results for education are highly similar and are shown in appendix 2 (p 7). Access to telehealth services, reasonable waiting times, and receipt of preventive services were unequal across multiple countries. In the countries that showed moderate access to telehealth (ie, Colombia, Peru, and Uruguay), access varied by more than 15 linear percentage points across income levels. Access to reasonable waiting times differed by more than 15 percentage points by income in these three countries. Other user experience outcomes differed by income in Mexico (respect, communication, and autonomy), Peru (communication and autonomy), and Uruguay (respect). Despite between-country difference in access to preventive services, all four countries showed strong within-country inequality by income (24 percentage points in Colombia to 35 percentage points in Mexico).

**Table 3:** Inequality in health system access and quality by income across four countries, slope index of inequality (95% CI)

	**Colombia**	**Mexico**	**Peru**	**Uruguay**
Quality source	0·04 (−0·07 to 0·15)	0·14 (0·01 to 0·28)	0·09 (−0·04 to 0·22)	0·06 (−0·06 to 0·19)
Telehealth	0·16 (0·05 to 0·27)	0·00 (−0·08 to 0·09)	0·16 (0·04 to 0·29)	0·20 (0·09 to 0·31)
Met chronic need	0·00 (−0·01 to 0·01)	0·06 (−0·08 to 0·20)	−0·07 (−0·16 to 0·03)	0·03 (−0·01 to 0·06)
Met mental health need	0·02 (−0·30 to 0·33)	0·07 (−0·23 to 0·36)	0·25 (0·05 to 0·45)	0·05 (−0·29 to 0·38)
Respect	0·02 (−0·12 to 0·17)	0·20 (0·04 to 0·35)	0·13 (−0·03 to 0·30)	0·21 (0·09 to 0·33)
Communication	0·06 (−0·08 to 0·19)	0·24 (0·09 to 0·40)	0·24 (0·08 to 0·39)	0·07 (−0·06 to 0·19)
Autonomy	0·03 (−0·09 to 0·16)	0·26 (0·12 to 0·41)	0·17 (0·02 to 0·32)	0·03 (−0·10 to 0·15)
Short waiting time	0·17 (0·04 to 0·30)	0·14 (−0·01 to 0·29)	0·23 (0·07 to 0·39)	0·22 (0·11 to 0·33)
Non-discrimination	0·09 (−0·03 to 0·20)	0·00 (−0·08 to 0·07)	0·01 (−0·12 to 0·14)	0·05 (−0·02 to 0·12)
Preventive check	0·24 (0·13 to 0·35)	0·35 (0·22 to 0·48)	0·27 (0·14 to 0·40)	0·21 (0·10 to 0·32)
Cardiovascular disease screening	−0·03 (−0·25 to 0·20)	0·14 (−0·07 to 0·35)	0·19 (−0·04 to 0·42)	0·06 (−0·11 to 0·23)
Continuity	−0·02 (−0·35 to 0·30)	0·34 (0·00 to 0·68)	0·21 (−0·13 to 0·56)	0·15 (−0·05 to 0·36)
Safety	0·09 (−0·02 to 0·20)	−0·05 (−0·12 to 0·02)	0·02 (−0·11 to 0·15)	0·10 (0·01 to 0·18)
Quality care	−0·10 (−0·21 to 0·01)	0·06 (−0·07 to 0·20)	−0·05 (−0·18 to 0·08)	0·26 (0·15 to 0·38)
Affordable care	0·02 (−0·11 to 0·15)	0·12 (0·00 to 0·24)	0·09 (−0·05 to 0·24)	0·38 (0·27 to 0·49)
Responsiveness	−0·15 (−0·27 to −0·03)	−0·17 (−0·29 to −0·05)	−0·10 (−0·25 to 0·04)	−0·10 (−0·23 to 0·02)
COVID-19 response	−0·15 (−0·26 to −0·05)	−0·03 (−0·16 to 0·10)	−0·08 (−0·21 to 0·05)	0·17 (0·05 to 0·29)

The magnitude of inequality required to attain statistical significance (ie, 95% CI excludes the null) is not constant across outcomes within country due to differences in the size of the relevant population as defined in table 1. A value of −1 indicates maximum pro-poor distribution; a value of +1 indicates maximum pro-wealthy distribution; 0 indicates equality.

Confidence in care quality and affordability and support for the government COVID-19 response was concentrated among wealthy respondents in Uruguay. Other confidence outcomes were not significantly unequal or were more likely among low-income respondents, particularly in Colombia.

Table 4 provides the results of the decomposition analysis for preventive checks, an outcome with significant income inequality across all four countries. Several patterns emerge: gender was an important component of income inequality, with women being more likely to have preventive service access than men in Colombia, Mexico, and Uruguay but less likely to report such access in Peru. Because women are less likely to be wealthy in all four countries, gender contributed negatively to income inequality in all countries except Peru. The contribution of education was inconsistent across countries, playing a larger role in Mexico (32% of inequality across incomes accounted for by university education) and Uruguay (22% contribution of secondary education to income inequality) than Colombia and Peru. After accounting for these individual characteristics, source of care explained little of the inequality in Mexico (private *vs* government sources accounts for 4·5% of observed inequality), moderate amounts in Colombia (16·7% for social security *vs* government care), and Peru (11·2% for private and 6·0% for no source compared with government care), and a great deal in Uruguay, where 89% of income inequality in preventive care was explained by usual source being within the social security sector. Substantial residual inequality is unexplained in this model (total percent contributions <100%).

**Table 4:** Decomposition of income inequalities in preventive care by demographics and health sector coverage

	**Colombia (n=1222; concentration index=0·16)**			**Mexico (n=928; concentration index=0·19)**			**Peru (n=1240; concentration index=0·16)**			**Uruguay (n=1213; concentration index=0·13)**		
	Elasticity	Contribution	Contribution to concentration index (%)	Elasticity	Contribution	Contribution to concentration index (%)	Elasticity	Contribution	Contribution to concentration index (%)	Elasticity	Contribution	Contribution to concentration index (%)
**Age, years (ref: 18–29)**												
30–39	−0·07	0·00	−1·2%	−0·05	0·00	−0·6%	−0·17	0·00	2·5%	−0·07	0·00	1·2%
40–49	−0·07	0·00	−0·6%	−0·06	0·00	0·7%	−0·14	0·00	1·8%	−0·02	0·00	−0·2%
50–59	−0·05	0·00	0·8%	−0·04	0·00	0·6%	−0·12	0·01	4·0%	−0·07	0·00	−0·8%
≥60	−0·05	0·01	4·7%	0·03	0·00	−0·7%	−0·01	0·00	−0·2%	−0·11	0·00	−3·5%
**Gender (ref: male)**												
Female	0·10	−0·02	−10·7%	0·03	−0·01	−4·2%	−0·12	0·02	14·7%	0·19	−0·05	−36·8%
**Education (ref: primary or less)**												
Secondary	0·04	0·00	3·0%	0·41	−0·02	−11·5%	−0·12	0·00	−0·1%	0·15	0·03	22·4%
Non–university	0·03	0·00	2·9%	0·01	0·00	0·0%	0·00	0·00	−0·1%	0·01	0·00	0·5%
University or higher	0·05	0·01	7·5%	0·20	0·06	32·2%	−0·01	0·00	−1·4%	0·05	0·01	7·9%
**Location (ref: urban)**												
Rural	−0·01	0·00	0·8%	−0·10	0·02	8·4%	−0·04	0·00	3·1%	−0·02	0·00	0·5%
Town or suburb	−0·07	0·01	6·0%	−0·05	0·01	3·6%	−0·04	0·01	4·1%	0·01	0·00	−0·8%
**Health status (ref: no chronic condition)**												
Chronic condition	−0·06	0·00	3·0%	0·01	0·00	−0·1%	−0·06	0·00	−0·4%	0·09	−0·01	−4·1%
**Usual source (ref: government)**												
None	−0·10	0·00	−0·4%	−0·04	0·00	−1·0%	−0·13	0·01	6·0%	−0·03	0·00	0·2%
Social security and military	0·07	0·03	16·7%	0·11	0·00	2·6%	0·03	0·00	2·1%	0·28	0·12	89·0%
Private	0·01	0·00	0·1%	0·07	0·01	4·5%	0·08	0·02	11·2%	0·05	0·01	4·2%

**Figure 2:**
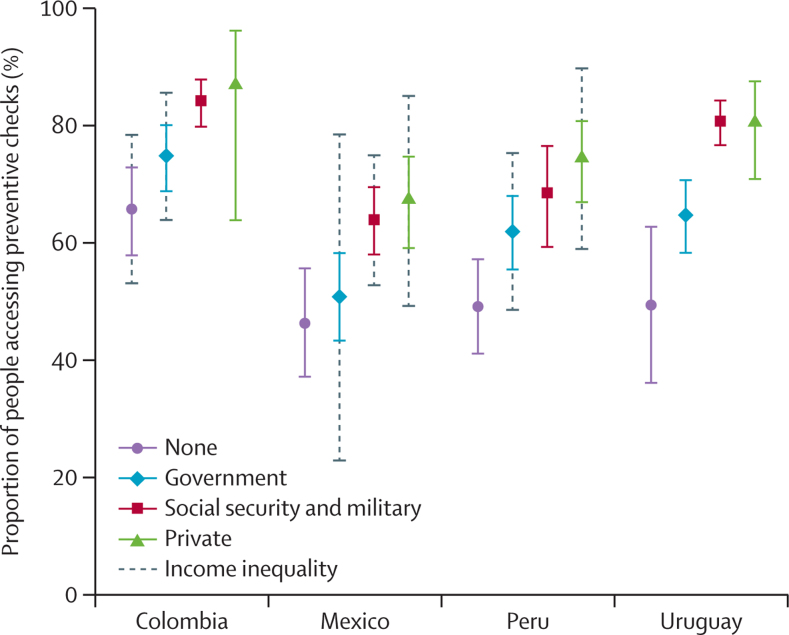
Prevalence and income inequality in access to preventive checks by usual source of care in Colombia, Mexico, Peru, and Uruguay Solid points and lines provide the proportion accessing preventive checks in the past year within each sector by country and the 95% CI around this estimate. Dashed lines represent the slope index of inequality centred around the proportion accessing preventive checks to indicate the magnitude of within-sector income inequality; only statistically significant (ie, p<0·05) slope index of inequality is shown.

Figure 2 provides context for interpreting these results. Access to preventive checks varied across care sources within each country, with an upward gradient from those without a usual source through government, social security, and, finally, the private sector. In Uruguay in particular, the receipt of preventive care services was markedly higher among people using social security and military facilities compared with government care or those with no usual source. Social security affiliates were wealthier than those reliant on government coverage (appendix 2 p 7), which is consistent across all study countries. However, within each sector in Uruguay, there was no statistically significant inequality in prevention checks by income (figure 2). The findings that prevention access was delivered consistently within the social security and military sector regardless of income, that this delivery is higher than the government services, and that those served by government facilities are generally on lower income contextualises the large role sector of care plays in inequality in Uruguay.

Colombia, Mexico, and Peru showed some significant income inequality within sectors, mostly notably in Mexico, where prevention check access differed by income within government, social security, and private sources (appendix 2 p 12). Although there were also differences between sectors and a concentration of lower-income respondents within government care for these three countries, the health-care sector itself contributed less to inequality in these countries than in Uruguay due to significant inequalities within the health-care sector.

## Discussion

This comprehensive assessment of health system quality across four countries in Latin America revealed substantial inequities between and within countries and had common implications across countries. Health-care use was high, with notable access to telehealth in three countries, showing innovations in care delivery in the wake of COVID-19. However, important health needs are unmet, particularly for mental health concerns. Telehealth access was unequal by income in countries with moderate access, whereas mental health needs were unmet across income groups within each country. Although user experience ratings were much lower in Peru than Mexico, they were significantly worse for the poorest respondents in both countries. Following the disruption of routine care due to the COVID-19 pandemic, access to basic preventive care varied across countries and between people with low or high incomes within countries.

Our results suggest that access to general health services is high in these countries, although access did not equate to high-quality access, needs for mental health-care access being met, or preventive care and screenings being provided. The long-standing scarcity of mental health resources in the region was accentuated by the COVID-19 pandemic.^32^, ^33^ Innovations, such as telehealth, offer promise in enhancing primary care access,^34^ but present a risk of worsening inequities given disproportionate access for wealthier individuals. Previous research attests to the extent of inequality in health coverage and access to care across the region.^35^ Our findings extend this knowledge to consider health system quality, identifying inequality in telehealth, in user experience domains, and in aspects of a competent health system, such as preventive care. Findings could also inform action in the wake of the COVID-19 pandemic.^14^, ^15^, ^16^, ^17^ We further identified country-specific contributions to observed inequality, including the low performance of the government care sector in Uruguay and both within-subsector and between-subsector inequality in the other countries.

Although comparisons between countries highlight gaps and strengths in health system quality, the four countries have varying socioeconomic and political contexts that should be considered when interpreting the results. Peru has experienced political upheaval in the last five years, resulting in inadequate investment in health-care infrastructure. We found an overall deficit in respect, autonomy, communication, non-discrimination, and safety in health care in Peru relative to other study countries. Despite the extension of social rights to health care, disadvantaged populations might be disproportionately affected by barriers that limit their access to adequate services and exacerbate financial hardship.^36^ Moreover, the pandemic has had a negative impact on several health indicators.^37^ In this context, government management of the COVID-19 pandemic was rated very poorly.

Respondents in Mexico expressed high confidence in the health system, despite the other outcomes being largely similar between countries. The most comparable study, a cross-national survey in 2013, also found higher health system confidence in Mexico than other countries in the region, although a less substantial difference was found.^38^, ^39^ These results contrast the difficulties that the health system has been facing during the reorganisation of care, with a decline in people with access to services mainly affecting people without employment benefits and living in rural areas.^40^ People with low socioeconomic status have the highest burden of disease, fewer protective factors, and receive less medical care.^41^ The situation is exacerbated by an increased number of people with low income and the financial crisis of social health-care institutions.^42^ Our finding of high confidence relative to other countries should be interpreted with caution given some differences in item wording, but might signify that survey respondents are confident despite changes or that those most affected by the changes were not fully captured in this survey.

Inequality is a constraint on Colombia's economic growth and social progress. Despite the country's social security system being based on the managed competition model, which led to increased coverage, some studies have found differences in the incidence, prevalence, and use of health services between the health affiliation schemes, and regional income inequality.^2^, ^43^, ^44^ Another study found no education-based disparities in health-care experience despite high income inequality.^35^ Putting these aspects in cross-national context, we found health system quality outcomes and levels of inequality largely similar to those in other study countries.

Among the countries studied, Uruguay stands out as the highest-income country with the most stable political environment and a better funded and better equipped health-care system. Several outcomes were higher in Uruguay across domains of system coverage, user experience, system competence (notably continuity of care), and handling of the COVID-19 pandemic, although this did not eliminate inequalities within the country. Wealthier individuals experienced greater confidence in the health system, and social security provides consistent access to prevention services regardless of income, with a higher standard of delivery than government care. Our findings coincided with a study that revealed evidence of inequity in terms of physician visits, medication usage, and non-access to health services due to financial barriers.^45^

We addressed four countries within a large region, which is not intended to represent Latin America as a whole. Within these countries, the use of mobile phone surveys excludes people without mobile phone access or who do not speak Spanish, under-representing individuals within the lowest socioeconomic status groups. Mobile phone coverage is high in these countries, we used a landline and mobile phone sample in Mexico to account for slightly lower mobile phone coverage, and we used post-stratification weighting to approximate the full population; nonetheless, findings might not fully capture the extent of health inequities and might overestimate outcomes such as the use of telehealth.

Regarding survey content, the translation of survey items and responses differed between Mexico and the other countries, which might have affected outcomes related to security, affordability, and responsiveness. Capturing health system use in a telephone survey and creating comparable indicators is challenging; respondents might not recall all visits or classify them correctly and substantial variance exists within categories used for analysis, including multiple types of private providers in a country (eg, Mexico) and distinctions between military and social security users and systems. Guidelines differ across countries; we applied a common standard for comparison, which might not match national policy precisely. In the analysis, the use of income classifications might have limited the precision of our analysis; although missing data was rare overall, 8% of respondents in Mexico declined to provide income information, further limiting inequality assessment. The assumption of a linear relationship to calculate the SIIs might not hold in all cases, and there might be non-linear patterns of health inequalities that were not captured. Observed predictors could not completely explain inequality in our decomposition analysis, with substantial unexplained residual variation making inference imperfect.

The study of health system quality reveals that, despite advances in care delivery, substantial inequities between and within countries persist. Examining health systems in four distinct countries enables benchmarking of multiple quality outcomes and identification of priorities nationally and regionally. Overall, our study underscores the importance of continued efforts to address the challenges facing the health-care systems in Latin America to ensure equitable access to high-quality care for all.

## Data sharing

Individual-level, de-identified data from the People's Voice Survey will be publicly available in mid-2024. Data will be available on the Harvard Dataverse. The survey instrument and data dictionary will be available upon publication.

## Declaration of interests

JMR declares having the role of assistant researcher at Cayetano Heredia University (San Martín de Porres, Peru). We declare no competing interests.
