# Supplementary material for: Inequalities in health system coverage and quality: a cross-sectional survey of four Latin American countries
Source: Lancet Glob Health. 2023 Dec 11;12(1):e145–55. doi: 10.1016/S2214-109X(23)00488-6 (PMC10716623; doi:10.1016/S2214-109X(23)00488-6)
Supplement: Supplementary appendix 2 [file mmc2.pdf]

THE LANCET
Global Health

Supplementary appendix 2

This appendix formed part of the original submission and has been peer reviewed. We post it as supplied by the authors.

Supplement to: Roberti J, Leslie HH, Doubova SV, et al. Inequalities in health system coverage and quality: a cross-sectional survey of four Latin American countries. *Lancet Glob Health* 2023; published online Dec 11. https://doi.org/10.1016/S2214-109X(23)00488-6.

Supplement A

Table 1: Example PVS items in English and Spanish translation

**Item**	**English**	**Southern American countries**	**Mexico**
**Usual source of care**	Is there one healthcare facility or healthcare provider's group you usually go to for most of your healthcare? Please do not include pharmacies, shops, or traditional healers.<br>Responses: Yes, No	¿Hay algún consultorio, clínica o establecimiento de salud al que acude la mayoría de las veces que necesita atención? Por favor no incluya farmacias, curanderos o medicina alternativa.<br>Responses: Sí, No	¿Hay algún establecimiento de salud o un grupo de profesionales de la salud a los que usted recurre la mayoría de las veces cuando necesita atención médica?<br>Responses: Sí, No
**Quality of usual source**	Overall, how would you rate the quality of healthcare you received in the past 12 months from this healthcare facility?<br><br>Response options<br>1. Excellent<br>2. Very good<br>3. Good<br>4. Fair<br>5. Poor<br>6. I did not receive healthcare from this provider in the past 12 months	En general, ¿cómo calificaría la calidad de atención que recibió en los últimos 12 meses en este establecimiento de salud?<br><br>Response options:<br>1. Excelente<br>2. Muy buena<br>3. Buena<br>4. Ni buena ni mala<br>5. Mala<br>6. No se atendió en ese lugar en los últimos 12 meses.	En general, ¿cómo calificaría la calidad de la atención que recibió en los últimos 12 meses en este establecimiento de salud?<br><br>Response options:<br>1 Excelente<br>2 Muy buena<br>3 Buena<br>4 Regular<br>5 Mala<br>6 No recibí atención médica en este centro en los últimos 12 meses
**Confidence items**			
**Quality care**	How confident are you that you would receive good quality healthcare if you became very sick?	¿Cuán seguro/a está de que, para una enfermedad grave, recibiría atención de buena calidad?	¿Hasta qué punto confía en que usted recibiría una atención de buena calidad si se enfermara gravemente?
**Affordable care**	How confident are you that you would be able to afford the healthcare you needed if you became very sick?	¿Cuán seguro/a está de que, para una enfermedad grave, podría pagar la atención que necesite?	¿Hasta qué punto confía en que usted podría pagar la atención médica que necesitara si se enfermara gravemente?
**Responsiveness**	How confident are you that the government considers the public's opinion when making decisions about the healthcare system?	¿Cuán seguro/a está de que se tiene en cuenta la opinión de la gente cuando se toman decisiones sobre las políticas de salud?	¿Hasta qué punto confía en que el Gobierno considera la opinión pública cuando toma decisiones sobre el sistema de salud?
**Response options**	1. Very confident<br>2. Somewhat confident	1. Muy seguro/a<br>2. Algo seguro/a	1. Confío mucho<br>2. Confío un poco

	3. Not too confident<br>4. Not at all confident	3. No muy seguro/a<br>4. Nada seguro/a	3. No confío mucho<br>4. No confío nada

Table 2: Cardiovascular screening guidelines

**Hypertension**	
Colombia	• Annually under 50 years old<br>• Every 3 months 50 and over
Mexico	• >20 years old with normal blood pressure: every 2-3 years<br>• >20 years old with elevated blood pressure: every 6 months
Peru	• >18 years old + no risk factors: every 5 years<br>• > 18 years old + risk factors: every year
Uruguay	• 18-39 years old + no risk factors: every 3 years<br>• 18-39 years old + risk factors: every year<br>• >40 years old: every year
International: European Society of Cardiology 2018	At every visit +<br>• Blood pressure <120/80: every 5 years<br>• Blood pressure 120-129/80-84: every 3 years<br>• Blood pressure 130-139/85-89: every year
**Diabetes mellitus**	
Colombia	Depending on FINDRISC score (>12), all patients >18 years old
Mexico	Depending on FINDRISC score (>9-12), all patients >18 years old
Peru	• <40 years old if family history of diabetes mellitus, gestational diabetes mellitus, polycystic ovary syndrome<br>• >40 years old if overweight or higher, or other risk factors (if normal, every 3 years)
Uruguay	• All ages with any risk factors: yearly<br>• Over 45 years old with no risk factors: every 3 years
International: American Diabetes Association 2023	<35 years old + 1 or more risk factors (including "Latino" as a risk factor) or >35 years old: minimum every 3 years or more frequently depending on test results and risk factors
**Cholesterol**	
Colombia	• Any age + risk factors<br>• >45 years old if no risk factors
Mexico	>20 years old
Peru	Any age with prior diagnosis of diabetes mellitus
Uruguay	• Men > 40 years old: every 2 years if no risk factors<br>• Postmenopausal women: every 2 years if no risk factors<br>• Adults with risk factors: annually
International: European Society of Cardiology 2019	• Men > 40 years old<br>• Women >50 years old or postmenopausal

Sources

International

- The Task Force for the management of arterial hypertension of the European Society of Cardiology (ESC) and the European Society of Hypertension (ESH): 2018 ESC/ESH Guidelines for the management of arterial hypertension

- The Task Force for the management of dyslipidaemias of the European Society of Cardiology (ESC) and European Atherosclerosis Society (EAS): 2019 ESC/EAS Guidelines for the management of dyslipidaemias: lipid modification to reduce cardiovascular risk
- 2021 ESC Guidelines on cardiovascular disease prevention in clinical practice, developed by the Task Force for cardiovascular disease prevention in clinical practice with representatives of the European Society of Cardiology and 12 medical societies
- ElSayed et al. Classification and Diagnosis of Diabetes: Standards of Care in Diabetes – 2023. American Diabetes Association

Colombian

- Sistema General de Seguridad Social en Salud –Colombia. 2014. Guía de práctica clínica para la prevención, detección temprana, diagnóstico, tratamiento y seguimiento de las dislipidemias en la población mayor de 18 años.
- Moya et al. 2018. Consenso de expertos sobre el manejo clínico de la hipertensión arterial en Colombia. Sociedad Colombiana de Cardiología y Cirugía.
- Aschner et al. 2016. Guía de práctica clínica para la prevención, diagnóstico, tratamiento y seguimiento de la diabetes mellitus tipo 2 en la población mayor de 18 años.

Mexican

- Pavía-López et al. 2022. Guía de práctica clínica mexicana para el diagnóstico y tratamiento de las dislipidemias y enfermedad cardiovascular aterosclerótica.
- Instituto Mexicano del Seguro Social Guía de práctica clínica:
    - Diagnóstico y tratamiento de hipertensión arterial en el adulto mayor 2017
    - Diagnóstico y tratamiento farmacológico de la Diabetes Mellitus Tipo 2 en El Primer Nivel de Atención 2016
    - Diagnóstico y tratamiento de dislipidemias (hipercolesterolemia) en el adulto 2016

Peruvian

- Ministerio de Salud, Guía de Práctica Clínica para el Diagnóstico, Tratamiento y Control de la Diabetes Mellitus Tipo 2 en el Primer Nivel de Atención 2016.
- Ministerio de Salud. Guía de Práctica Clínica para el Diagnóstico, Tratamiento y Control de la Enfermedad Hipertensiva 2015.
- Ministerio de Salud. Guía de Práctica Clínica para el Diagnóstico, Manejo y Control de Dislipidemia, Complicaciones Renales y oculares en Personas con Diabetes Mellitus Tipo 2 2017.

Uruguayan

- Ministerio de Salud Pública. Recomendaciones para el abordaje de la hipertensión arterial sistémica en el primer nivel de atención. 2017
- Ministerio de Salud. Guía de práctica clínica de diabetes mellitus tipo 2 para la atención en el ámbito ambulatorio. 2017.
- Ministerio de Salud. Guía Nacional para el abordaje de las dislipemias en el adulto. 2019.

Table 3: Income and education classifications and respondent distributions (weighted)

**Colombia (N=1237)**			
**Monthly household income**		**Education**	
No income	174 (14.2%)	None	48 (3.8%)
< 75,000 pesos	92 (7.5%)	Preschool	62 (5.0%)
75,000 to 200,000 pesos	121 (9.9%)	Elementary school	503 (40.6%)
200,000 to 400,000 pesos	134 (11.0%)	Secondary	360 (29.0%)
400,000 to 600,000 pesos	138 (11.2%)	Non-university superior	137 (11.0%)
600,000 to 800,000 pesos	151 (12.3%)	University superior	105 (8.5%)
800,000 to 10M pesos	211 (17.3%)	Postgraduate	26 (2.1%)

> 10M pesos	202 (16.6%)		
**Mexico (N=1002)**			
**Monthly household income**		**Education**	
< 6,500 pesos	538 (58.2%)	None	38 (3.7%)
6,500-10,000 pesos	193 (20.8%)	Primary	258 (25.7%)
10,000-15,000 pesos	89 (9.6%)	Secondary	298 (29.7%)
15,000-25,000 pesos	65 (6.9%)	Preparatoria o Bachillerato	235 (23.4%)
> 25,000 pesos	42 (4.5%)	Technical career	17 (1.6%)
		University	138 (13.8%)
		Postgraduate	22 (2.1%)
**Peru (N=1255)**			
**Monthly household income**		**Education**	
No income	66 (5.3%)	None	2 (0.2%)
< S/.1.000	571 (46.2%)	Initial	68 (5.4%)
S/. 1.000 – 2.500	409 (33.1%)	Primary	409 (32.6%)
S/. 2.501 – 3.500	92 (7.4%)	Secondary	563 (44.9%)
S/. 3.501 – 5.500	46 (3.7%)	Non-university higher education	78 (6.2%)
S/. 5.501 – 7.500	23 (1.8%)	Higher University	114 (9.1%)
S/. 7.501 – 10.000	17 (1.3%)	Postgraduate	23 (1.8%)
> S/.10.000	15 (1.2%)		
**Uruguay (N=1237)**			
**Monthly household income**		**Education**	
No income	12 (1.0%)	None	3 (0.2%)
1,200 pesos or less	24 (2.0%)	Initial/preschool	23 (1.8%)
1,200 to 14,000 pesos	191 (15.7%)	Elementary	523 (42.3%)
14,000 to 30,000 pesos	320 (26.3%)	Secondary (basic cycle and 4th to 6th)	537 (43.4%)
30,000 to 40,000 pesos	201 (16.5%)	Non-university higher	36 (2.9%)
40,000 to 50,000 pesos	122 (10.0%)	University superior	106 (8.5%)
50,000 to 65,000 pesos	84 (6.9%)	Postgraduate	11 (0.9%)
> 65,000 pesos	264 (21.7%)		

Table 4: Classification of respondents into sectors by country

	**None**	**Government**	**Social security and Military**	**Private**
**Colombia**				
Coverage	N/A	Subsidized<br>SISBEN<br>None	Contributory special<br>Contributory prepaid	Private (particular)
Source of care for last visit (for user experience and continuity outcomes)	No in-person visit within 12/months	At least one visit AND coverage = Subsidized OR SISBEN OR None	At least one visit AND coverage = Contributory special OR Contributory prepaid	At least one visit AND coverage = Private
Usual source of care (for all other outcomes)	No usual source of care	Yes usual source AND coverage = Subsidized OR SISBEN OR None	Yes usual source AND coverage = Contributory special OR	Yes usual source AND coverage = Private

			Contributory prepaid	
**Mexico**				
Coverage	NA	IMSS-Bienestar (previously *Seguro Popular* and INSABI)<br>None	Social security (IMSS) or Social Security for State Workers (ISSSTE)<br>State Petroleum (PEMEX) or Military	Private (particular, pharmacy)
Source of care for last visit (for user experience and continuity outcomes)	No in-person visit within 12/months	At least one visit AND last visit facility ownership = IMSS-Bienestar (previously *Seguro Popular*) OR Secretaria de Salud o Servicios Estatales de Salud	At least one visit AND last visit facility ownership = IMSS OR ISSSTE OR PEMEX, Defensa o Marina	At least one visit AND last visit facility ownership = Private
Usual source of care (for all other outcomes)	No usual source of care	Usual source of care AND usual source ownership = IMSS-Bienestar (antes Seguro Popular) OR Secretaria de Salud o Servicios Estatales de Salud	Usual source of care AND usual source ownership = IMSS OR ISSSTE OR PEMEX, Defensa o Marina	Usual source of care AND usual source ownership = Private
**Peru**				
Coverage	None	SIS (Seguro Integral de Salud)	EsSalud<br>Sanidad de las Fuerzas Armadas y Policiales	Private
Source of care for last visit (for user experience and continuity outcomes)	No in-person visit within 12/months	At least one visit AND last visit facility ownership = Public AND coverage = SIS OR None	At least one visit AND last visit facility ownership = Public AND coverage = EsSalud OR Sanidad de las Fuerzas Armadas y Policiales	At least one visit AND last visit facility ownership = Private
Usual source of care (for all other outcomes)	No usual source of care	Usual source of care AND usual source ownership = Public AND coverage = SIS OR None OR	Usual source of care AND usual source ownership = Public AND coverage = EsSalud OR	Usual source of care AND usual source ownership = PRIVATE

		Refused OR private	Sanidad de las Fuerzas Armadas y Policiales	
**Uruguay**				
Coverage	NA	State Health Service Administration (ASSE) / University Hospital	Mutual Police / Military Hospital	Private
Source of care for last visit (for user experience and continuity outcomes)	No in-person visit within 12/months	At least one visit AND last visit facility ownership = Public	At least one visit AND last visit facility ownership = Mutual	At least one visit AND last visit facility ownership = Private
Usual source of care (for all other outcomes)	No usual source of care	Usual source of care AND usual source ownership = Public	Usual source of care AND usual source ownership = Mutual	Usual source of care AND usual source ownership = Private

Box 1: Erreygers concentration index for binary outcomes

$$CI = 4\mu_h \left(\frac{2}{N^2}\mu_h \sum h_i R_i\right)$$

N = number of observations
R = deviation of rank from mean or median rank socioeconomic status
h = health outcome
i = individual
μ_h = mean of h

Table 5: Call outcomes by country

**Call Outcomes**	**Category**	**Colombia**	**Mexico**	**Peru**	**Uruguay**
		N	N	N	N
Total sample		10,425	54,161	33,294	18,132
Interviews	I (eligible)	1,237	1002	1,255	1,237
Interviews - failed quality control		42	0	36	48
Refusal	R (eligible)	3,519	1724	4,987	6,214
Other non-response	O (eligible)	146	0	12	4,502
Non-Contact	UE (unknown eligibility)	5,206	45,484	24,327	4,692
Not eligible	NE (not eligible)	275	5951	2,677	1,439

Table 6: Slope Index of Inequalities in health system outcomes by educational status

**Outcome**	Colombia	Mexico	Peru	Uruguay
	SII (95% CI)	SII (95% CI)	SII (95% CI)	SII (95% CI)
Quality source	0.06 (-0.05, 0.17)	**0.23 (0.11, 0.35)**	-0.10 (-0.23, 0.04)	0.02 (-0.10, 0.14)
Telehealth	**0.23 (0.11, 0.34)**	0.01 (-0.07, 0.09)	**0.24 (0.11, 0.37)**	**0.26 (0.15, 0.37)**
Met chronic need	-0.01 (-0.03, 0.02)	0.18 (-0.11, 0.48)	-0.14 (-0.29, 0.01)	0.01 (-0.03, 0.06)
Met mental health need	-0.27 (-0.57, 0.03)	0.06 (-0.19, 0.31)	0.19 (-0.03, 0.42)	0.18 (-0.13, 0.50)
Respect	-0.09 (-0.24, 0.05)	**0.42 (0.29, 0.54)**	0.07 (-0.11, 0.24)	**0.12 (0.00, 0.24)**
Communication	-0.02 (-0.16, 0.12)	**0.38 (0.25, 0.51)**	-0.12 (-0.30, 0.07)	0.11 (-0.01, 0.23)
Autonomy	0.03 (-0.11, 0.16)	**0.44 (0.33, 0.56)**	0.04 (-0.13, 0.21)	0.01 (-0.12, 0.13)
Short wait	**0.19 (0.05, 0.33)**	**0.24 (0.11, 0.37)**	0.09 (-0.08, 0.26)	0.08 (-0.03, 0.20)
Non-discrimination	0.00 (-0.08, 0.09)	-0.02 (-0.09, 0.05)	-0.05 (-0.15, 0.06)	0.06 (-0.01, 0.13)
Preventive check	**0.22 (0.11, 0.34)**	**0.41 (0.30, 0.52)**	0.09 (-0.06, 0.23)	**0.28 (0.17, 0.39)**
CVD screening	0.01 (-0.21, 0.23)	0.13 (-0.05, 0.32)	0.12 (-0.11, 0.36)	0.11 (-0.05, 0.28)
Continuity	-0.05 (-0.36, 0.26)	**0.43 (0.16, 0.69)**	0.19 (-0.18, 0.55)	0.08 (-0.14, 0.30)
Safety	0.08 (-0.04, 0.21)	**-0.07 (-0.13, -0.01)**	-0.04 (-0.15, 0.07)	-0.02 (-0.10, 0.05)
Quality care	-0.11 (-0.23, 0.01)	0.06 (-0.07, 0.18)	-0.14 (-0.28, 0.00)	**0.16 (0.04, 0.27)**
Affordable care	-0.03 (-0.16, 0.11)	0.10 (-0.02, 0.21)	-0.04 (-0.20, 0.12)	**0.23 (0.11, 0.34)**
Responsiveness	**-0.16 (-0.28, -0.03)**	**-0.13 (-0.25, -0.02)**	**-0.26 (-0.41, -0.11)**	-0.06 (-0.18, 0.06)
COVID response	**-0.17 (-0.28, -0.06)**	-0.01 (-0.13, 0.1)	**-0.24 (-0.39, -0.09)**	0.11 (-0.01, 0.23)

Table 7: Characteristics of Respondents by Usual Source of Care
A: Colombia

	None (N = 267)	Subsidized (N = 460)	Contributory (N = 469)	Private (N = 25)	p-value
**Age**					0.528
18-29	87 (32.0%)	126 (24.3%)	124 (30.5%)	10 (32.1%)	
30-39	56 (20.6%)	106 (20.5%)	91 (22.3%)	9 (29.1%)	
40-49	41 (14.9%)	96 (18.6%)	59 (14.3%)	2 (6.0%)	
50-59	48 (17.8%)	72 (13.8%)	64 (15.7%)	7 (23.9%)	
>= 60	40 (14.8%)	118 (22.8%)	70 (17.1%)	3 (8.8%)	
**Gender**					0.028
Male	144 (53.3%)	223 (43.2%)	195 (48.3%)	21 (73.6%)	
Female	126 (46.4%)	293 (56.7%)	209 (51.7%)	8 (26.4%)	
Another gender	1 (0.3%)	1 (0.1%)	0 (0.0%)	0 (0.0%)	
**Rural, town, city location**					<0.001
Rural	35 (12.6%)	101 (19.6%)	31 (7.6%)	8 (26.9%)	
Town/suburb	64 (23.6%)	175 (33.9%)	89 (21.9%)	11 (35.5%)	
City	173 (63.8%)	240 (46.6%)	285 (70.5%)	11 (37.6%)	
**Education**					<0.001
Primary or less	125 (46.2%)	344 (66.6%)	122 (30.0%)	13 (42.3%)	
Secondary	89 (32.9%)	127 (24.5%)	129 (31.6%)	10 (33.7%)	
Non-university tertiary	30 (10.8%)	30 (5.7%)	75 (18.4%)	4 (12.0%)	
University or more	28 (10.1%)	17 (3.2%)	82 (20.0%)	4 (12.0%)	

	None (N = 267)	Subsidized (N = 460)	Contributory (N = 469)	Private (N = 25)	p-value
**Income**					<0.001
Lowest income	83 (31.3%)	221 (43.5%)	70 (17.4%)	6 (20.4%)	
Middle income	97 (36.5%)	200 (39.2%)	108 (26.7%)	10 (34.5%)	
Highest income	85 (32.2%)	89 (17.3%)	225 (55.9%)	13 (45.1%)	
**Health coverage**					
Subsidized	162 (61.3%)				
Contributory	94 (35.6%)				
Private	9 (3.1%)				
**Chronic illness**					0.002
No	221 (81.7%)	356 (69.0%)	288 (70.9%)	26 (88.3%)	
Yes	50 (18.3%)	160 (31.0%)	119 (29.1%)	4 (11.7%)	
**Total health care visits past 12 months**					<0.001
Non-user	82 (30.3%)	52 (10.0%)	24 (5.8%)	6 (20.8%)	
Occasional user (1-4)	136 (50.1%)	288 (55.6%)	201 (49.5%)	16 (55.7%)	
Frequent user (>4)	53 (19.6%)	178 (34.4%)	182 (44.7%)	7 (23.5%)	

B: Mexico

	None (N = 153)	Government (N = 236)	Social security/military (N = 391)	Private (N = 209)	p-value
**Age**					0.001
18-29	66 (34.7%)	57 (21.9%)	86 (24.1%)	66 (34.6%)	
30-39	41 (21.7%)	58 (22.5%)	69 (19.2%)	41 (21.2%)	
40-49	38 (20.3%)	62 (24.0%)	65 (18.2%)	26 (13.3%)	
50-59	22 (11.4%)	41 (15.7%)	58 (16.2%)	27 (14.1%)	
>= 60	23 (11.8%)	41 (16.0%)	80 (22.3%)	32 (16.7%)	
**Gender**					0.041
Male	100 (52.9%)	102 (39.6%)	180 (50.4%)	90 (47.4%)	
Female	85 (45.3%)	155 (60.4%)	177 (49.6%)	100 (52.6%)	
Another gender	4 (1.8%)	0 (0.0%)	0 (0.0%)	0 (0.0%)	
**Rural, town, city location**					<0.001
Rural	47 (25.2%)	78 (30.5%)	59 (16.7%)	32 (16.5%)	
Town/suburb	68 (36.7%)	105 (40.9%)	133 (37.7%)	58 (30.8%)	
City	71 (38.1%)	73 (28.5%)	161 (45.6%)	100 (52.7%)	
**Education**					<0.001
Primary or less	72 (38.1%)	90 (34.9%)	82 (23.0%)	47 (24.5%)	
Secondary	86 (45.9%)	151 (58.8%)	207 (58.2%)	82 (43.1%)	
Non-university tertiary	2 (0.9%)	3 (0.9%)	9 (2.3%)	5 (2.1%)	
University or more	29 (15.1%)	14 (5.4%)	59 (16.5%)	58 (30.3%)	
**Income**					<0.001
Lowest income	82 (47.5%)	187 (78.1%)	181 (54.6%)	81 (46.8%)	
Middle income	60 (34.8%)	31 (12.9%)	74 (22.1%)	28 (15.8%)	
Highest income	31 (17.7%)	22 (9.1%)	78 (23.3%)	65 (37.4%)	
**Health coverage**					<0.001
Government	80 (46.6%)	192 (79.7%)	38 (10.8%)	71 (40.4%)	

	None (N = 153)	Government (N = 236)	Social security/military (N = 391)	Private (N = 209)	p-value
Social Security and Military	75 (43.6%)	39 (15.9%)	306 (87.4%)	62 (35.4%)	
Private	17 (9.8%)	11 (4.4%)	7 (1.8%)	43 (24.3%)	
**Chronic illness**					0.026
No	154 (82.7%)	197 (76.8%)	261 (73.3%)	142 (74.7%)	
Yes	33 (17.3%)	60 (23.2%)	95 (26.7%)	49 (25.3%)	
**Total health care visits past 12 months**					<0.001
Non-user	80 (42.5%)	59 (22.8%)	49 (13.6%)	13 (6.7%)	
Occasional user (1-4)	72 (38.1%)	113 (44.1%)	153 (43.0%)	111 (58.4%)	
Frequent user (>4)	37 (19.4%)	85 (33.1%)	155 (43.4%)	67 (34.9%)	

C: Peru

	None (N = 277)	Government (N = 438)	Social security/military (N = 232)	Private (N = 307)	p-value
**Age**					<0.001
18-29	85 (28.3%)	152 (30.2%)	34 (16.1%)	59 (23.8%)	
30-39	78 (25.9%)	124 (24.7%)	32 (14.9%)	58 (23.6%)	
40-49	62 (20.5%)	103 (20.4%)	33 (15.6%)	42 (17.0%)	
50-59	40 (13.2%)	66 (13.2%)	33 (15.5%)	34 (13.8%)	
>= 60	37 (12.1%)	59 (11.6%)	80 (37.9%)	54 (21.8%)	
**Gender**					0.019
Male	188 (62.8%)	196 (39.0%)	110 (52.1%)	135 (55.2%)	
Female	112 (37.2%)	306 (61.0%)	101 (47.9%)	109 (44.5%)	
Another gender	0 (0.0%)	0 (0.0%)	0 (0.0%)	1 (0.3%)	
**Rural, town, city location**					<0.001
Rural	69 (23.0%)	108 (21.5%)	26 (12.3%)	21 (8.3%)	
Town/suburb	45 (14.9%)	123 (24.4%)	23 (10.7%)	31 (12.3%)	
City	186 (62.0%)	271 (54.1%)	162 (77.0%)	195 (79.4%)	
**Education**					<0.001
Primary or less	117 (39.0%)	229 (45.6%)	54 (26.0%)	77 (31.3%)	
Secondary	144 (47.9%)	233 (46.4%)	87 (41.8%)	101 (41.2%)	
Non-university tertiary	18 (5.9%)	23 (4.5%)	20 (9.2%)	19 (7.6%)	
University or more	22 (7.2%)	18 (3.5%)	48 (23.0%)	49 (19.9%)	
**Income**					<0.001
Lowest income	168 (56.2%)	327 (66.7%)	68 (32.5%)	73 (30.6%)	
Middle income	93 (30.9%)	130 (26.6%)	97 (46.2%)	91 (37.8%)	
Highest income	39 (12.8%)	33 (6.7%)	45 (21.3%)	76 (31.6%)	
**Health coverage**					<0.001
Uninsured	100 (33.3%)	72 (14.3%)	0 (0.0%)	44 (17.9%)	
Government	119 (39.5%)	424 (84.8%)	0 (0.0%)	86 (35.2%)	
Social Security and Military	79 (26.3%)	0 (0.0%)	210 (100.0%)	81 (33.2%)	
Private	3 (1.0%)	5 (0.9%)	0 (0.0%)	34 (13.8%)	

	None (N = 277)	Governmen t (N = 438)	Social security/mil itary (N = 232)	Private (N = 307)	p-value
**Chronic illness**					0.016
No	248 (82.6%)	395 (78.8%)	134 (63.8%)	167 (68.3%)	
Yes	52 (17.4%)	107 (21.2%)	76 (36.2%)	78 (31.7%)	
**Total health care visits past 12 months**					<0.001
Non-user	113 (37.7%)	49 (9.6%)	32 (15.0%)	26 (10.4%)	
Occasional user (1-4)	139 (46.4%)	276 (54.9%)	72 (34.2%)	130 (53.0%)	
Frequent user (>4)	48 (15.8%)	178 (35.4%)	107 (50.8%)	90 (36.6%)	

D: Uruguay

	None (N = 82)	Government (N = 357)	Social security/military (N = 663)	Private (N = 135)	p-value
**Age**					0.181
18-29	16 (19.1%)	122 (27.3%)	98 (16.3%)	33 (28.1%)	
30-39	29 (34.4%)	84 (18.9%)	121 (20.3%)	16 (13.9%)	
40-49	15 (17.2%)	72 (16.1%)	97 (16.2%)	20 (17.3%)	
50-59	12 (13.5%)	59 (13.2%)	95 (15.9%)	23 (19.9%)	
>= 60	14 (15.7%)	109 (24.5%)	186 (31.3%)	25 (20.9%)	
**Gender**					0.689
Male	44 (52.6%)	187 (42.1%)	298 (50.0%)	62 (53.6%)	
Female	39 (46.4%)	257 (57.7%)	296 (49.7%)	54 (46.4%)	
Another gender	1 (1.1%)	1 (0.2%)	2 (0.3%)	0 (0.0%)	
**Rural, town, city location**					0.007
Rural	10 (11.4%)	41 (9.2%)	40 (6.7%)	3 (2.4%)	
Town/suburb	5 (5.2%)	62 (14.0%)	59 (9.8%)	6 (4.7%)	
City	68 (83.5%)	339 (76.8%)	495 (83.4%)	108 (92.8%)	
**Education**					<0.001
Primary or less	41 (49.2%)	269 (60.4%)	204 (34.4%)	34 (29.3%)	
Secondary	36 (42.5%)	163 (36.5%)	279 (46.9%)	62 (53.0%)	
Non-university tertiary	3 (2.8%)	4 (0.7%)	28 (4.6%)	4 (2.8%)	
University or more	5 (5.5%)	11 (2.4%)	84 (14.1%)	18 (14.9%)	
**Income**					<0.001
Lowest income	35 (43.2%)	326 (74.6%)	163 (27.7%)	24 (20.5%)	
Middle income	31 (38.9%)	82 (18.6%)	176 (30.0%)	35 (30.1%)	
Highest income	15 (17.9%)	30 (6.8%)	248 (42.2%)	56 (49.3%)	
**Health coverage**					<0.001
Government	36 (44.1%)	400 (90.2%)	11 (1.8%)	6 (4.9%)	
Social Security and Military	36 (44.9%)	43 (9.5%)	558 (94.5%)	60 (51.8%)	
Private	9 (11.0%)	2 (0.3%)	22 (3.6%)	50 (43.3%)	
**Chronic illness**					0.016
No	61 (72.5%)	243 (54.7%)	328 (55.1%)	73 (62.5%)	
Yes	23 (27.5%)	202 (45.3%)	268 (44.9%)	44 (37.5%)	
**Total health care visits past 12 months**					<0.001
Non-user	28 (33.0%)	58 (13.0%)	24 (4.0%)	9 (7.1%)	
Occasional user (1-4)	30 (35.2%)	199 (44.6%)	222 (37.2%)	47 (40.1%)	
Frequent user (>4)	27 (31.8%)	189 (42.4%)	350 (58.8%)	61 (52.9%)	

Supplemental Figure 2: Health system outcomes between and income inequality within sub-sectors across 4 countries

Legend: Figures below provide the estimated prevalence and 95% confidence interval for each health system coverage and quality outcome by subsector. Users are classified by subsector as shown in Table 3 above; classification is based on usual source of care for all outcomes except for items asked specifically of the most recent visit (respect, communication, autonomy, short-wait, and continuity), where classification is based on source of care. Individuals without an in-person visit in the past 12 months are excluded from items related to the most recent visit; confidence intervals could not be calculated for small samples with no variance in outcomes.

Dashed lines depict slope index of inequality by income group within subsector centred on prevalence of the outcome for that group; SII with significance $p<0.05$ are shown.

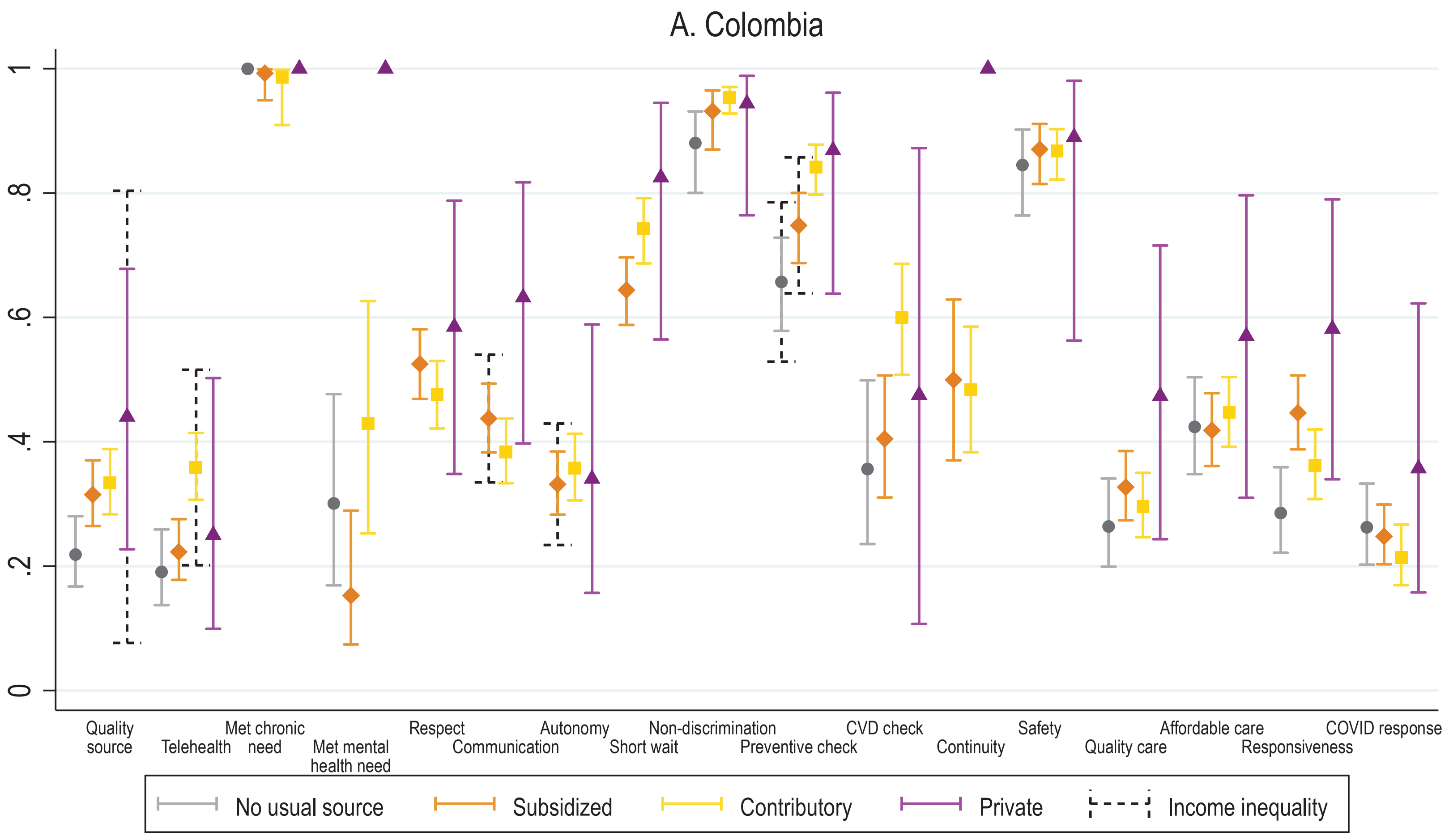
A. Colombia
0
.2
.4
.6
.8
1
Quality source
Telehealth
Met chronic need
Met mental health need
Respect
Communication
Autonomy
Short wait
Non-discrimination
Preventive check
CVD check
Continuity
Safety
Quality care
Affordable care
Responsiveness
COVID response
No usual source
Subsidized
Contributory
Private
Income inequality

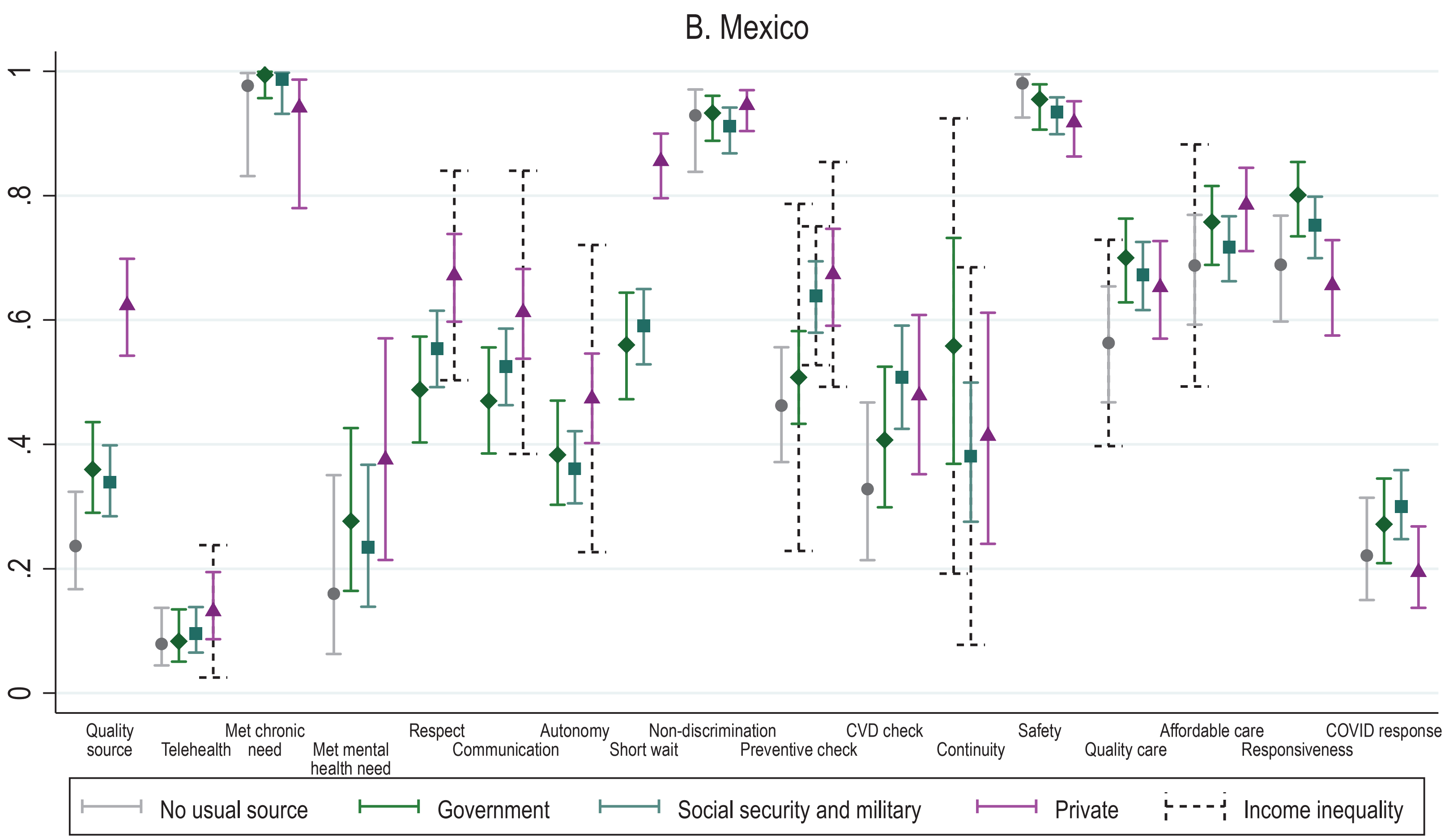
B. Mexico
0
.2
.4
.6
.8
1
Quality source
Telehealth
Met chronic need
Met mental health need
Respect
Communication
Autonomy
Short wait
Non-discrimination
Preventive check
CVD check
Continuity
Safety
Quality care
Affordable care
Responsiveness
COVID response
No usual source
Government
Social security and military
Private
Income inequality

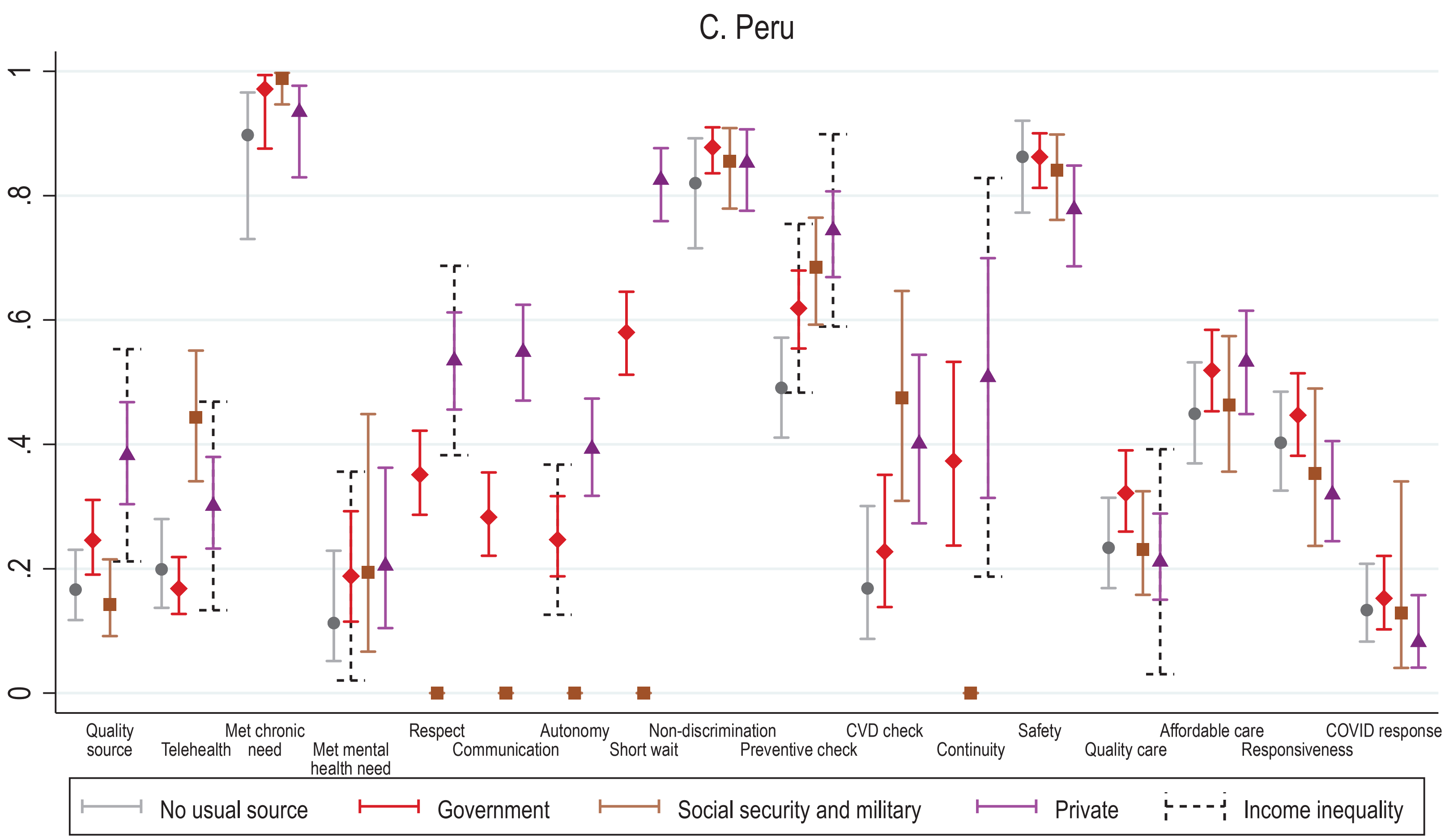
C. Peru
0
.2
.4
.6
.8
1
Quality source
Telehealth
Met chronic need
Met mental health need
Respect
Communication
Autonomy
Short wait
Non-discrimination
Preventive check
CVD check
Continuity
Safety
Quality care
Affordable care
Responsiveness
COVID response
No usual source
Government
Social security and military
Private
Income inequality

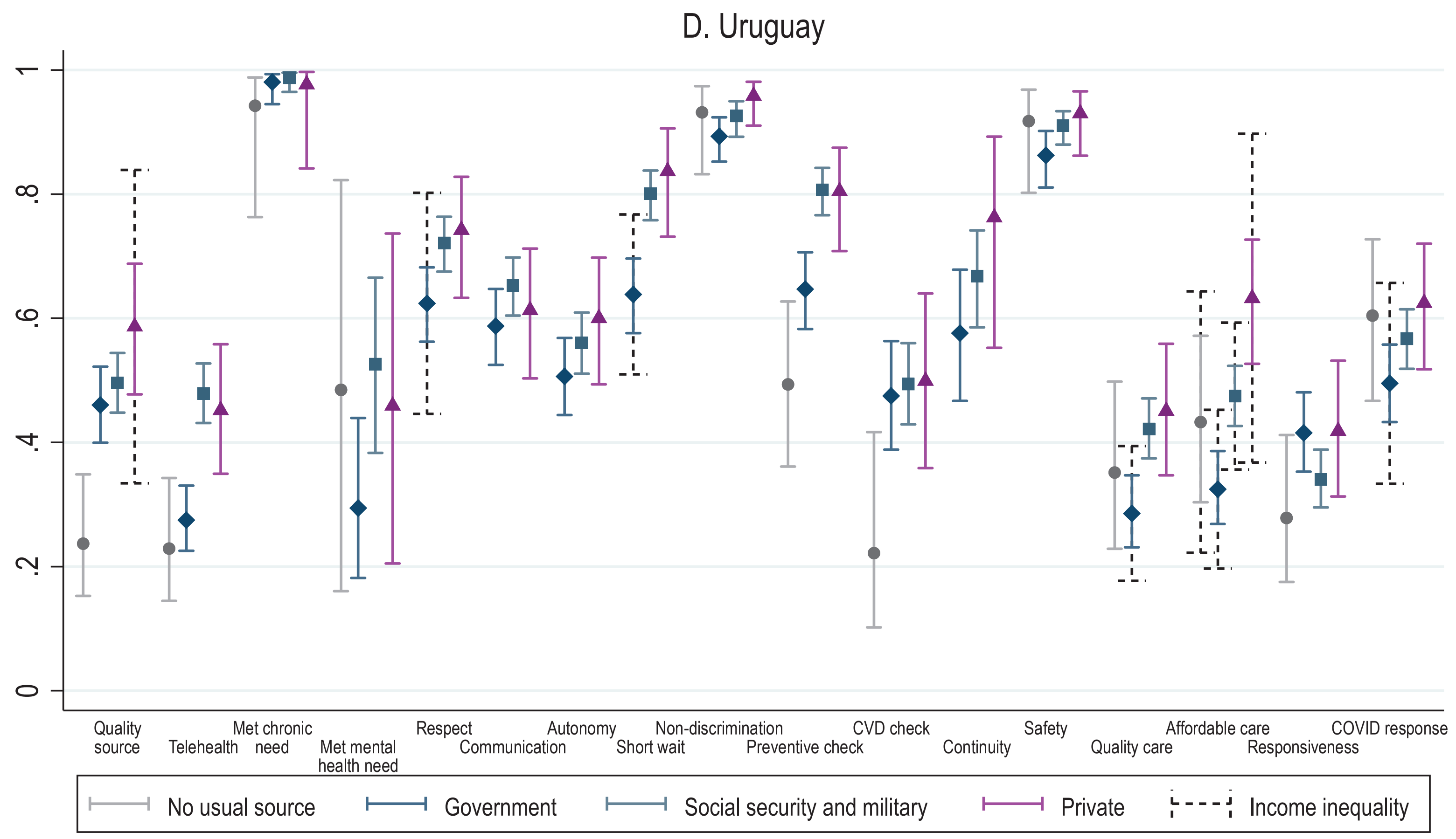
D. Uruguay
1
.8
.6
.4
.2
0
Quality source
Telehealth
Met chronic need
Met mental health need
Respect
Communication
Autonomy
Short wait
Non-discrimination
Preventive check
CVD check
Continuity
Safety
Quality care
Affordable care
Responsiveness
COVID response
No usual source
Government
Social security and military
Private
Income inequality

Supplement C

HACER QUE SE ESCHUCHE LA “VOZ DE LA GENTE” SOBRE LA CALIDAD DEL SISTEMA DE SALUD EN AMÉRICA LATINA EN TIEMPOS DE CRISIS

FINANCIADO POR:
Banco Interamericano de Desarrollo

INVESTIGADORES
Investigador País:
Dra. Patricia J. García Funegra
Universidad Peruana Cayetana Heredia, Lima, Perú

Co-Investigadores
Dra. Hannah Leslie
Harvard T.H. School of Public Health, Boston, Estados Unidos

Dr. Ezequiel García Elorrio
Instituto de Efectividad Clínica y Sanitaria, Buenos Aires, Argentina

Dra. Brittany Blouin
Universidad Peruana Cayetana Heredia, Lima, Perú

Médico - Cirujano Jesús Guillermo Medina Ranillla
Universidad Peruana Cayetana Heredia, Lima, Perú

Versión 1.3

6 de mayo del 2021

Resumen Ejecutivo: Hacer que se escuche la "voz de la gente" sobre la Calidad del Sistema de Salud en América Latina en Tiempos de Crisis

Nombre en inglés: *Making the People´s Voice Heard on Health System Quality in Latin America in Times of Crisis*

Las emergencias de salud pública, incluida la pandemia de COVID-19, han puesto de manifiesto importantes deficiencias en los sistemas de salud de todo el mundo, especialmente en países de ingresos bajos y medianos. La calidad del sistema de salud es imprescindible para mejorar la salud de la población y la confianza en el sistema. Se carece de instrumentos rápidos y comparables para medir la calidad de los sistemas de salud y estos instrumentos son esenciales para mejorar los sistemas de salud. Es por ello que la Red QuEST (Quality Evidence for Health System Transformation – Evidencia de Calidad para la Transformación de los Sistemas de Salud), una red global de expertos en calidad de atención con base en la Universidad de Harvard, ha estado trabajando este último año en el desarrollo de instrumentos que pudieran servir para este fin. Estos nuevos instrumentos deberían permitirnos "escuchar la Voz de la Gente" en aspectos como 1) razones de la utilización y la no utilización del sistema de salud, 2) comparación de las percepciones de la calidad del sistema de salud entre países y entre grupos dentro de un mismo país, 3) identificar qué partes del sistema de salud actualmente responden a necesidades críticas de la población, y 4) evaluar la relación entre las percepciones de la calidad del cuidado y la confianza en el sistema de salud.

Uno de los instrumentos desarrollados es la encuesta "Voz de la Gente"- People's Voice Survey (PVS), que será implementada en los países de la Red QuEST. La encuesta PVS será validada localmente en cada país. En Latinoamérica se administrará a una muestra representativa a nivel nacional de adultos en Colombia, México, Perú y Uruguay. La encuesta medirá las características de la población, el uso de los servicios de salud, los procesos de atención de salud recibidos y la confianza en el sistema de salud. Se administrará a nivel nacional, por teléfono, a un total de 4000 personas por país en dos rondas, a finales del 2021 y en el 2023.

El otro instrumento propuesto es la encuesta "Voz de la Gente"- cohortes electrónicas (e-Cohortes), que se desarrollará posteriormente en base a la experiencia y los resultados obtenidos con la encuesta PVS.

Este protocolo y los instrumentos para la encuesta PVS ya cuentan con la aprobación del Comité de Ética de la Universidad de Harvard (ver ANEXO 1, 2 y 3). Como miembros de la Red QuEST Latinoamérica, participaremos en (1) la validación local de los instrumentos y (2) la aplicación de las encuestas. Con este protocolo, estamos solicitando la aprobación del Comité de Ética para las actividades relacionadas al PVS y en una segunda fase, aplicaremos a la revisión ética de los instrumentos y actividades que se desarrollarán para las e-Cohortes.

1. Antecedentes

El acceso al cuidado de la salud se está expandiendo globalmente debido a que los países tienen por objetivo el logro de la Cobertura Universal de Salud (CUS). Sin embargo, el acceso no siempre se traduce en un mejor cuidado: cinco millones de personas mueren cada año por condiciones tratables a pesar de haber accedido al sistema de salud.[1] Estos números pueden incrementarse ya que cada vez más personas buscan el cuidado de su salud y la carga de enfermedad cambia a condiciones más complejas. La mortalidad materna y neonatal en muchos países se ha estancado en niveles por encima de los deseados, a pesar de grandes esfuerzos, mostrando que las estrategias actuales no son suficientes. Las estrategias actuales para mejorar la calidad a nivel micro no son adecuadas,[3,4] por lo que el sistema de salud en su totalidad debe ser transformado para lograr reducir la mortalidad. Estos hallazgos fueron delineados en la *Lancet Global Health Commission on High Quality Health Systems* y repercutieron ampliamente entre los países.[2] La pandemia de COVID-19 resaltó aún más la importancia crítica del buen funcionamiento del sistema de salud, de manera tal que pueda responder al problema sin descuidar el resto de la atención. Para lograr crear sistemas de salud con estas características, es necesario realizar investigaciones a gran escala y traducción de políticas. Por ejemplo, es esencial aprender del momento actual para lograr desarrollar y probar un rango de modelos de atención de la salud que a) sean útiles para las necesidades específicas de los pacientes con COVID-19, y b)

continúen brindando servicios de alta calidad en forma segura para el resto de la población.

Esta transformación a gran escala necesita mediciones que sean comparables en el tiempo y entre países para medir y evaluar el cambio. En este momento existen pocos instrumentos rápidos y comparables que midan el desempeño del sistema de salud. Los proyectos existentes para medir la calidad del sistema de salud en países de bajos y medianos ingresos incluyen evaluaciones de usuarios del sistema en determinadas instituciones de salud (como encuestas al egreso del paciente [5]) y evaluaciones periódicas de corte transversal de instituciones de salud [6] o encuestas a la población realizadas una única vez.[7,8] Es necesaria la realización de evaluaciones a nivel poblacional sobre la calidad del sistema de salud y sobre las preferencias de los usuarios que puedan realizarse en forma rápida, repetida en el tiempo, y que permita la comparación entre países. Las encuestas **"Voz de la Gente",** incluyendo las encuestas **PVS (encuesta transversal)** y **e-Cohortes (encuesta de seguimiento),** se han planteado como una forma de cerrar estas brechas.

2. Justificación

Las encuestas **"Voz de la Gente"** proveerán información de varios países en relación a cómo los individuos utilizan el sistema de salud y cómo califican el cuidado de su salud en el contexto de la pandemia de COVID-19. La encuesta PVS permitirá evaluar la utilización del sistema de salud, diagnosticar las razones más importantes de la no-utilización del sistema de salud, comparar la calidad del cuidado entre sectores o países y la confianza en el sistema de salud en su totalidad. En el caso de las e-Cohortes, que se planean dirigir a poblaciones con enfermedades crónicas, nos permitirá acercarnos al manejo y seguimiento en el tiempo y la calidad de la atención en estas poblaciones.

Teniendo en cuenta que el proceso de desarrollo de estas encuestas incluye la participación de colaboradores de los distintos países y consultas a decisores, informantes clave y población objetivo, los hallazgos de la misma serán útiles para la evaluación de programas y toma de decisiones para mejorar el acceso y la calidad del cuidado de la salud. A través de la Red QuEST **(***Quality Evidence for Health System*

Transformation – Evidencia de Calidad para la Transformación de los Sistemas de Salud), los métodos y hallazgos de las encuestas “Voz de la Gente” se convertirán en bienes públicos para maximizar el impacto en la comunidad de investigadores en sistemas de salud y decisores a nivel mundial. Comprender mejor las experiencias de cuidado de la salud de la población, particularmente en el contexto de la pandemia, será de utilidad para inversiones futuras a fin de mejorar la calidad de los sistemas de salud de manera global.

3. Objetivos

3.1 Objetivo General

Validar localmente y aplicar las encuestas **“Voz de la Gente”** para evaluar diversos aspectos de la calidad de atención en servicios de salud, y recoger data que pueda permitir comparabilidad con otros países.

3.2 Objetivos específicos

1. Determinar la utilización y la no-utilización del sistema de salud
2. Comparar las percepciones de la calidad del sistema de salud – experiencia del usuario y calidad del cuidado entre diversos grupos.
3. Identificar qué partes del sistema de salud actualmente responden a necesidades críticas de la población.
4. Evaluar la relación entre las percepciones de la calidad del cuidado y la confianza en el sistema de salud.
5. Evaluar la experiencia prospectiva y longitudinal (e-Cohortes), desde el reporte de los pacientes, de los resultados y la calidad de la atención sanitaria para las personas con enfermedades crónicas.

4. Diseño del estudio y métodos

La investigación propuesta está conformada por 2 estudios: 1) La encuesta "Voz de la Gente"- People's Voice Survey (**PVS**) y 2) La encuesta "Voz de la Gente"- cohortes electrónicas (**e-Cohortes**). Una descripción detallada de la metodología para la encuesta PVS se proporciona en las secciones siguientes, así como una breve descripción del estudio de las e-Cohortes al final de esta sección. La metodología detallada para el estudio de las e-Cohortes será presentada para su aprobación correspondiente al Comité de Ética cuando esté completamente desarrollada, antes de iniciar con el estudio.

4.1 Encuesta de corte transversal denominada "Voz de la Gente" – People's Voice Survey (PVS).

4.1.1 Población de estudio

El estudio completo se llevará a cabo en cuatro países, incluyendo Colombia, México, Perú y Uruguay. En cada país, la población objetivo son adultos de 18 años o más, hombres y mujeres, usuarios y no usuarios del sistema de salud, con acceso a telefonía. Los criterios de inclusión incluyen: 1) Tener 18 años o más, 2) Tener acceso a un teléfono nacional y 3) Contar con su consentimiento para participar en el estudio. No existen criterios de exclusión.

4.1.2 Diseño del estudio

Se trata de un estudio de corte transversal, en dos etapas o momentos en el tiempo: **Ola 1**, cuya recolección de datos se realizará en la segunda mitad del año 2021, y **Ola 2**, cuya recolección de datos será en la primera mitad del año 2023. La encuesta PVS ha sido desarrollada por un grupo colaborativo de expertos que incluye la Red QuEST Global (compuesta por grupos en América Latina, África y Asia) con el objetivo de medir: características de la población, uso de servicios de salud, procesos del cuidado de salud recibido y confianza en el sistema de salud (ver ANEXO 3).

4.1.3 Revisión de experiencias nacionales de encuestas de calidad en salud

Se realizará una búsqueda bibliográfica exhaustiva, incluyendo literatura gris y sitios web, sobre experiencias previas en encuestas que evalúen la calidad de atención en salud en los usuarios, en cada uno de los países en los que se aplicarán los instrumentos presentados.

4.1.4 Validación local de la encuesta

El objetivo de esta fase es traducir y adaptar la encuesta al sistema de salud nacional y evaluar la claridad y relevancia de cada pregunta. Se usará la última versión de la encuesta aprobada por el Comité de Ética de Harvard (ver ANEXO 3).

4.1.4.1 Traducción de la encuesta

Miembros del equipo de investigación realizarán la traducción al español y traducción reversa al inglés para asegurar la calidad, claridad y equivalencia de la traducción. Se adaptará la encuesta en español respecto a las palabras /fraseos que sean más comunes en cada uno de los países, así como a las características demográficas (p. ej. idiomas hablados) y tipos de servicios de salud propios para cada país.

4.1.4.2 Evaluación cognitiva de la encuesta

El propósito de esta etapa es evaluar el desempeño de cada una de las preguntas de la encuesta PVS con relación al lenguaje, confiabilidad del entendimiento y completitud de las opciones de respuesta. Se realizarán 5-10 entrevistas cognitivas por país. Dadas las circunstancias de la pandemia, se optará por un muestreo por conveniencia a personas mayores de 18 años, tratando de incluir diversas características: ambos sexos, distintos grupos etarios, nivel socioeconómico y uso de servicios de salud públicos y privados. Una vez identificados los potenciales participantes, se les explicará por vía telefónica o por videoconferencia en línea el objetivo del estudio. Si estuviesen interesados, se les leerá la Hoja Informativa que se adjunta (ver ANEXO 4) y se procederá a programar la fecha de la entrevista cognitiva. De ser requerido por el participante, se le puede enviar la Hoja Informativa por correo electrónico.

Estas entrevistas se llevarán a cabo por teléfono o videoconferencia en línea y tendrán una duración de 1 hora a 1 hora y media. En la metodología de la entrevista cognitiva (ver ANEXO 5), se pedirá a los participantes que "piensen en voz alta" (*think aloud*) mientras responden a la encuesta PVS para evaluar cómo entienden las preguntas propuestas. Se les pedirá luego que respondan, según sea necesario, a preguntas predeterminadas para explorar la comprensión de las preguntas de la encuesta, el proceso mental que siguieron para llegar a su respuesta y verificar si las opciones de respuesta brindadas son adecuadas (este proceso se llama "sondeo"). No todas las preguntas de la encuesta PVS serán evaluadas de manera cognitiva, solo aquellas en las cuales se identifique que pudiera haber problemas de interpretación.

Las entrevistas cognitivas serán grabadas con el permiso de los participantes, y si no se concediera dicho permiso, se tomarían notas en físico o en un archivo virtual. Los hallazgos clave transcritos serán guardados de manera anónima y analizados por el equipo de investigación para mejorar la encuesta PVS. Las respuestas no se analizarán como parte de los objetivos de la investigación. Una vez que los cambios propuestos sean implementados en la encuesta PVS por los equipos de investigación en cada país, en coordinación con el Grupo QuEST Global, los hallazgos de las entrevistas cognitivas y las grabaciones serán descartados.

4.1.5 Prueba piloto de la encuesta PVS

Se hará un piloto de la encuesta PVS a través de llamadas telefónicas, incluyendo a 50 encuestados por país. Se utilizará la misma metodología que para la encuesta final (ver siguiente sección) incluyendo una Hoja Informativa para el consentimiento en la que se señala que esto es un piloto y que los datos no se usarán en el análisis final (ver ANEXO 6). Los resultados se analizarán para garantizar la coherencia y claridad de las preguntas y para informar cualquier modificación a los instrumentos o a los procedimientos. Una vez los cambios propuestos sean implementados en la encuesta PVS final por los equipos de investigación en cada país en coordinación con el Grupo QuEST Global, los hallazgos de la prueba piloto de la encuesta serán descartados.

4.1.6 Encuesta PVS

Una vez finalizada la validación y el piloto de los instrumentos, se realizarán dos olas de la encuesta en los países mencionados. La encuesta PVS es una encuesta telefónica. La recolección de datos será llevada a cabo por una empresa privada y externa a la institución de investigación en cada uno de los países. Esta debe poseer experiencia en conducir encuestas telefónicas, respetando las regulaciones locales. Por cada ola, la empresa de recopilación de datos realizará una marcación de dígitos aleatorios, correspondientes a números de teléfono móviles o fijos, de acuerdo con las regulaciones aplicables en cada país. Las llamadas conectadas serán invitadas a participar, consentidas y evaluadas. Un breve guion presentará la encuesta y solicitará el Consentimiento Informado verbal para participar (ver ANEXO 7). Se obtendrá el consentimiento verbal y no se recopilará ninguna firma, ya que toda la interacción se llevará a cabo por teléfono. A los participantes que den su consentimiento se les preguntará si pueden completar la encuesta en ese momento o si prefieren programar otra oportunidad para devolver la llamada. En cualquier caso, los participantes pueden omitir cualquier pregunta o pueden colgar en cualquier momento durante la llamada. La empresa encuestadora registrará las respuestas de forma anónima, de manera que sea imposible rastrear a los participantes por sus datos o por su número de teléfono. La encuesta tomará aproximadamente 20-40 minutos. Para propósitos de asegurar la calidad de la encuesta, se elegirá al azar un 10% de estas y se grabarán fragmentos de las mismas para monitoreo.

4.1.7 Tamaño muestral

En nuestro país, se reclutará un total de 4000 participantes en el estudio, con 2000 participantes reclutados en la Ola 1 (en 2021) y 2000 participantes reclutados en la Ola 2 (en 2023).

Este tamaño muestral está basado en la experiencia de encuestas representativas a nivel nacional e internacionalmente comparables como Afrobarometer, Latinobarómetro o encuestas recientes utilizadas en respuesta a la pandemia de COVID-19.[9-11] Una muestra de 2000 participantes permite un margen de error de muestreo de ± 2.2 % para estimaciones de la muestra completa, como la proporción de encuestados que utilizan

la atención primaria en el primer contacto con el sistema de salud para sus necesidades de salud.

Hay una probabilidad del 90% de que las calificaciones de calidad medidas del 1 al 10 tengan un intervalo de confianza al 95% de 1 punto de amplitud para cualquier subconjunto de la población con al menos 100 encuestados (asumiendo una desviación estándar de 2.5 como un estimado conservador). Esto puede representar la calificación de la competencia de un proveedor de servicios de salud, entre varones con una visita reciente a un establecimiento de salud, o la calificación de la respuesta del sistema de salud contra el COVID-19, entre encuestados que reportan haber requerido cuidados de salud para tratamiento o testeo de COVID-19.

Una muestra de 2000 participantes proporciona un poder de 82% para detectar una diferencia de proporciones de 6.5% entre 2 grupos iguales, tal como encuestados varones y mujeres, aún en una proporción del 50%.

4.1.8 Análisis estadístico

Los datos se analizarán mediante estadística descriptiva y regresión lineal. Stata y R se utilizarán para el análisis y la presentación gráfica de los resultados.

4.2 Encuesta de corte longitudinal denominada "Voz de la Gente"- cohortes electrónicas (e-Cohortes)

Los detalles específicos de la metodología del estudio de e-Cohortes serán desarrollados teniendo en cuenta la experiencia de la Ola 1 de la encuesta PVS. Una vez esta metodología haya sido desarrollada completamente, será presentada para la aprobación correspondiente por el Comité de Ética antes de comenzar el estudio.

Se planea usar el estudio de e-Cohortes para evaluar las experiencias de los pacientes en el sistema de salud, longitudinalmente, con respecto al manejo de las enfermedades crónicas no transmisibles. Los adultos diagnosticados con una enfermedad crónica no transmisible específica (p. ej. hipertensión arterial o diabetes mellitus) serán invitados a participar. Cuestionarios mensuales serán administrados por teléfono o

videoconferencia en línea para medir el uso reciente de servicios de salud, experiencias con los servicios de salud y el estado de salud actual. Se estima incluir entre 800-1200 participantes que serían reclutados en 2022 y seguidos a lo largo de 1 año. Se planea realizar, antes de la aplicación de esta encuesta, un proceso de validación (traducción del instrumento y entrevistas cognitivas) y un piloto.

5. Aspectos éticos

Para la encuesta PVS se contará con 3 tipos de consentimientos: 1) Para las entrevistas cognitivas, la Hoja Informativa de la evaluación cognitiva de la encuesta PVS (ver ANEXO 4), 2) Para el estudio piloto, la Hoja Informativa del piloto de la encuesta PVS (ver ANEXO 6) y 3) Para la encuesta final, el Consentimiento Informado verbal para la encuesta PVS (ver ANEXO 7). El protocolo y los instrumentos a usarse serán presentados a los comités de ética correspondientes de cada país participante. Todos los instrumentos que usaremos en el PVS han sido aprobados por el Comité de Ética de Harvard en su versión en inglés (ver ANEXO 1, 2 y 3).

Hay dos riesgos potenciales de participar en la encuesta PVS. El primero podría ser el de la violación de la privacidad/identidad y el segundo la posible angustia emocional por responder preguntas sobre una experiencia de atención médica reciente. Consideramos que el primer riesgo es mínimo o nulo dado que las preguntas no son sensibles y las opciones de respuesta están diseñadas para que los encuestados no necesiten dar ninguna información personal. Se pide a los encuestados que respondan preguntas sobre sus experiencias y opiniones sobre la atención médica y su salud autoevaluada en general, pero no otros detalles sobre su estado de salud. Con respecto al segundo riesgo potencial de experimentar cierta angustia emocional al recordar sus experiencias para nuestra encuesta, consideramos que esta angustia sería poco probable, leve y de corta duración. La encuesta está diseñada para recopilar información mínimamente sensible. Para minimizar la angustia emocional de este estudio, no hemos hecho preguntas detalladas sobre los diagnósticos de los encuestados.

Los participantes pueden optar por que se les vuelva a llamar para completar la encuesta en el momento que elijan, para que puedan asegurarse de tener el tiempo y la privacidad adecuados para responder. Los participantes pueden omitir preguntas o colgar en cualquier momento. Los datos se mantendrán sin identificadores individuales.

Dado que este proyecto se trabajará con una encuestadora privada, en los términos de referencia del contrato con la misma, además de considerar la normativa de la protección de datos personales según la legislación peruana, se incluirá el desarrollo de una sesión de entrenamiento por parte del grupo de investigación. Este entrenamiento tendrá como objetivo capacitar a los encuestadores sobre: los objetivos de la investigación, aspectos éticos y conducta responsable en investigación, confidencialidad, el proceso de toma de consentimiento informado y el proceso de toma de datos de la encuesta.

La base de datos será almacenada por la empresa de recopilación de datos. Los datos se entregarán al equipo de estudio en un formato completamente anónimo. Los datos se almacenarán en computadoras protegidas con contraseña. Los datos serán analizados por personas expresamente comprometidas con la investigación y conocedoras de los protocolos de confidencialidad. Sólo los datos de la encuesta PVS final serán almacenados hasta el fin del estudio; como se describe en las secciones anteriores, los datos obtenidos para la fase de validación (entrevistas cognitivas), en el estudio piloto y los utilizados para la evaluación de la calidad de la entrevista (PVS final) serán eliminados una vez hayan cumplido su propósito.

6. Transferencia de conocimiento y difusión

Los resultados se compartirán a través de varias publicaciones revisadas por pares. Los resultados de la encuesta se publicarán en manuscritos para realizar comparaciones entre países, así como en análisis específicos de cada país dirigidos por los investigadores del país. Se publicará un resumen para el público en general y para los responsables de la formulación de políticas en el sitio web de la Red QuEST y se compartirá directamente con los colaboradores de QuEST. Anticipamos que los

resultados del estudio de investigación propuesto se comunicarán eficazmente a las comunidades participantes a través de estos medios.

7. Cronograma de trabajo

	2021										2022				2023				2024	
**Actividades**	Marzo	Abril	Mayo	Junio	Julio	Agosto	Septiembre	Octubre	Noviembre	Diciembre	1er Trimestre	2º Trimestre	3er Trimestre	4to Trimestre	1er Trimestre	2º Trimestre	3er Trimestre	4to Trimestre	1er Trimestre	2º Trimestre
Desarrollo del protocolo	✓	✓																		
Revisión y aprobación ética del protocolo general y de la metodología de la encuesta PVS			✓	✓																
Revisión bibliográfica de experiencias previas en calidad en salud				✓																
Validación de la encuesta PVS (traducción y entrevistas cognitivas)				✓	✓															
Encuesta PVS piloto					✓	✓														
Recolección de datos de la encuesta PVS Ola 1							✓	✓	✓	✓										
Recolección de datos de la encuesta PVS Ola 2															✓	✓				

Análisis y diseminación de datos de la encuesta PVS											✓	✓	✓	✓			✓	✓	✓	
Desarrollo de la encuesta e-Cohortes											✓									
Revisión y aprobación ética de la metodología de la encuesta e-Cohortes											✓									
Validación y piloto de la encuesta e-Cohortes												✓								
Recolección de datos de la encuesta e-Cohortes													✓	✓	✓	✓	✓			
Análisis y diseminación de datos de la encuesta e-Cohortes																		✓	✓	✓

8. Presupuesto

Presupuesto	
Desarrollo de la encuesta PVS	$ 12,863.85
Evaluación cognitiva de la encuesta PVS	$ 4,983
Piloto de la encuesta PVS	$ 5,200
Análisis y reporte de la encuesta PVS Ola 1	$ 22,000
Análisis y reporte de la encuesta PVS Ola 2	$ 19,000
Desarrollo de la encuesta e-Cohortes	$ 16,467
Evaluación cognitiva de la encuesta e-Cohortes	$ 4,500
Piloto de la encuesta e-Cohortes	$ 9,854
Recolección de datos de encuesta e-Cohortes	$ 34,667
Análisis y reporte de la encuesta e-Cohortes	$ 34,667
Sub-total	$164,201.85
Overhead (8%)	$13,136.15
**TOTAL**	**$ 177,338**

9. Referencias bibliográficas

1. Kruk ME, Gage AD, Joseph NT, Danaei G, García-Saisó S, Salomon JA. Mortality due to low-quality health systems in the universal health coverage era: a systematic analysis of amenable deaths in 137 countries. *The Lancet*. Elsevier; 2018 Nov 17;**392**(10160):2203–2212. [Citado 2021 Abr 30]. Disponible en: https://www.thelancet.com/journals/lancet/article/PIIS0140-6736(18)31668-4/fulltext

2. Rowe AK, Rowe SY, Peters DH, Holloway KA, Chalker J, Ross-Degnan D. Effectiveness of strategies to improve health-care provider practices in low-income and middle-income countries: a systematic review. *The Lancet Global Health*. 2018 Nov 1;**6**(11):e1163–e1175. [Citado 2021 Abr 30]. Disponible en: https://www.thelancet.com/journals/langlo/article/PIIS2214-109X(18)30398-X/fulltext

3. Semrau KEA, Hirschhorn LR, Marx Delaney M, et al. Outcomes of a Coaching-Based WHO Safe Childbirth Checklist Program in India. *New England Journal of Medicine*. 2017 Dec 14;**377**(24):2313–2324. [Citado 2021 Abr 30]. Disponible en: https://www.nejm.org/doi/10.1056/NEJMoa1701075

4. Kruk ME, Gage AD, Arsenault C, et al. High-quality health systems in the Sustainable Development Goals era: time for a revolution. *The Lancet Global Health*. 2018 Nov 1;**6**(11):e1196–e1252. [Citado 2021 Abr 30]. Disponible en: https://www.thelancet.com/journals/langlo/article/PIIS2214-109X(18)30386-3/fulltext

5. Mucha T, Marilyn J. Acceso a medicamentos para controlar la hipertensión arterial prescritos en consulta externa en las Instituciones Prestadoras de Servicios de Salud (IPRESS) en Perú, ENSUSALUD 2016. *Universidad Peruana Cayetano Heredia* [Internet]. 2019 [Citado 2020 Ene 15]. Disponible en: http://repositorio.upch.edu.pe/handle/upch/6611

6. Leslie HH, Sun Z, Kruk ME. Association between infrastructure and observed quality of care in 4 healthcare services: A cross-sectional study of 4,300 facilities in 8 countries. *PLOS Medicine*. 2017 Dec 12;**14**(12):e1002464. [Citado 2021 Abr 30]. Disponible en: https://journals.plos.org/plosmedicine/article?id=10.1371/journal.pmed.1002464

7. Roder-DeWan S, Gage AD, Hirschhorn LR, et al. Expectations of healthcare quality: A cross-sectional study of internet users in 12 low- and middle-income countries. *PLOS Medicine*. Public Library of Science; 2019 Aug 7;**16**(8):e1002879. [Citado 2021 Abr 30]. Disponible en: https://journals.plos.org/plosmedicine/article?id=10.1371/journal.pmed.1002879

8. Guanais F, Doubova SV, Leslie HH, Perez-Cuevas R, García-Elorrio E, Kruk ME. Patient-centered primary care and self-rated health in 6 Latin American and Caribbean countries: Analysis of a public opinion cross-sectional survey. *PLOS Medicine*. 2018 Oct 9;**15**(10):e1002673. [Citado 2021 Abr 30]. Disponible en: https://journals.plos.org/plosmedicine/article?id=10.1371/journal.pmed.1002673

9. Afrobarometer. Sampling principles and weighting [Internet] [Citado 2021 Abr 30]. Disponible en : https://afrobarometer.org/surveys-and-methods/sampling-principles

10. Latinobarómetro. Latinobarómetro Database. [Internet] [Citado 2021 Abr 30]. Disponible en: https://www.latinobarometro.org/latContents.jsp

11. Ipsos. Responding to COVID-19 in Africa: Key findings from surveys in 20 countries. [Internet] [Citado 2021 Abr 30]. Disponible en: https://www.ipsos.com/en/responding-covid-19-africa-key-findings-from-surveys-in-20-countries

10. Anexos

ANEXO 1: Carta de aprobación del Comité de Ética de la Universidad de Harvard

ANEXO 2: Instrumentos aprobados en inglés por el Comité de Ética de la Universidad de Harvard

ANEXO 3: Encuesta PVS en inglés (última versión)

ANEXO 4: Hoja Informativa de la evaluación cognitiva

ANEXO 5: Guía de evaluación cognitiva (para el entrevistador)

ANEXO 6: Hoja Informativa del piloto de la encuesta PVS

ANEXO 7: Consentimiento Informado verbal para la encuesta PVS
